# Eco‐evolutionary responses to recreational fishing under different harvest regulations

**DOI:** 10.1002/ece3.4270

**Published:** 2018-09-11

**Authors:** Daniel Ayllón, Steven F. Railsback, Ana Almodóvar, Graciela G. Nicola, Simone Vincenzi, Benigno Elvira, Volker Grimm

**Affiliations:** ^1^ Faculty of Biology Department of Biodiversity, Ecology and Evolution Complutense University of Madrid Madrid Spain; ^2^ Department of Ecological Modelling Helmholtz Centre for Environmental Research – UFZ Leipzig Germany; ^3^ Department of Mathematics Humboldt State University Arcata California; ^4^ Lang Railsback & Associates Arcata California; ^5^ Department of Environmental Sciences University of Castilla‐La Mancha Toledo Spain; ^6^ Institute of Marine Sciences University of California Santa Cruz Santa Cruz California

**Keywords:** brown trout, eco‐evolutionary dynamics, eco‐genetic modeling, fishery‐induced evolution, harvest regulations, individual‐based model, recreational fisheries management

## Abstract

Harvesting alters demography and life histories of exploited populations, and there is mounting evidence that rapid phenotypic changes at the individual level can occur when harvest is intensive. Therefore, recreational fishing is expected to induce both ecological and rapid evolutionary changes in fish populations and consequently requires rigorous management. However, little is known about the coupled demographic and evolutionary consequences of alternative harvest regulations in managed freshwater fisheries. We used a structurally realistic individual‐based model and implemented an eco‐genetic approach that accounts for microevolution, phenotypic plasticity, adaptive behavior, density‐dependent processes, and cryptic mortality sources (illegal harvest and hooking mortality after catch and release). We explored the consequences of a range of harvest regulations, involving different combinations of exploitation intensity and minimum and maximum‐length limits, on the eco‐evolutionary trajectories of a freshwater fish stock. Our 100‐year simulations of size‐selective harvest through recreational fishing produced negative demographic and structural changes in the simulated population, but also plastic and evolutionary responses that compensated for such changes and prevented population collapse even under intense fishing pressure and liberal harvest regulations. Fishing‐induced demographic and evolutionary changes were driven by the harvest regime, and the strength of responses increased with increasing exploitation intensity and decreasing restriction in length limits. Cryptic mortality strongly amplified the impacts of harvest and might be exerting a selective pressure that opposes that of size‐selective harvest. “Slot” limits on harvestable length had overall positive effects but lower than expected ability to buffer harvest impacts. Harvest regulations strongly shape the eco‐evolutionary dynamics of exploited fish stocks and thus should be considered in setting management policies. Our findings suggest that plastic and evolutionary responses buffer the demographic impacts of fishing, but intense fishing pressure and liberal harvest regulations may lead to an unstructured, juvenescent population that would put the sustainability of the stock at risk. Our study also indicates that high rates of cryptic mortality may make harvest regulations based on harvest slot limits ineffective.

## 1 INTRODUCTION

Contemporary evolution is driven by several anthropogenic pressures that often operate on the same traits and thus impose new adaptive challenges to populations (Sullivan, Bird, & Perry, 2017). Harvest is a well‐documented example (Allendorf, England, Luikart, Ritchie, & Ryman, 2008). Intensive and size‐selective harvest of the individuals with the highest fitness potential exerts a strong directional selection force on fitness‐related traits in exploited populations (Allendorf et al., 2008; Cameron, O'Sullivan, Reynolds, Piertney, & Benton, 2013; Ernande, Dieckmann, & Heino, 2004; Fenberg & Roy, 2008; Uusi‐Heikkilä et al., 2015). Harvest induces phenotypic changes at rates several times greater than those from natural and other anthropogenic disturbances (Darimont et al., 2009). In particular, there is evidence that fishing causes changes to numerous population‐level ecological attributes and interrelated phenotypic expressions at the individual level (Sharpe & Hendry, 2009; Uusi‐Heikkilä et al., 2015; Wang & Höök, 2009). There is little doubt that evolution induced by commercial and recreational fisheries is taking place in exploited fish stocks, which might affect population viability, productivity, and recovery when slowly reversible, or even irreversible, genetic changes are involved (Enberg, Jørgensen, Dunlop, Heino, & Dieckmann, 2009; Fenberg & Roy, 2008; Jørgensen et al., 2007; Kuparinen & Merilä, 2007; Laugen et al., 2014). Numerous modeling, experimental, and empirical studies have assessed the evolutionary consequences of intensive, size‐selective fishing on physiological, behavioral, and life history traits and the resulting effects for population dynamics and fishery yields (e.g., Dunlop, Heino, & Dieckmann, 2009; Edeline et al., 2007; Heino, 1998; Piou, Taylor, Papaïx, & Prévost, 2015). However, analyses of fishing‐induced evolution in inland recreational fisheries are scarce (but see Thériault, Dunlop, Dieckmann, Bernatchez, & Dodson, 2008; Arlinghaus, Matsumura, & Dieckmann, 2009; Saura et al., 2010; Matsumura, Arlinghaus, & Dieckmann, 2011; Sutter et al., 2012), and management of such fisheries rarely considers the consequences of evolution.

Recreational fisheries now constitute the dominant or sole use of wild freshwater fish stocks in most industrialized countries and are becoming an important economic resource in many emerging economies and developing countries (Arlinghaus et al., 2017). The few previous studies indicate that recreational fishing might substantially alter the eco‐evolutionary trajectories of fish stocks. The systematic removal of large fish leads to changes in the size and age structure of fish stocks by truncating the population distribution and reduces abundance of reproductive age classes (Almodóvar & Nicola, 2004; Almodóvar, Nicola, & Suárez, 2002; Hixon, Johnson, & Sogard, 2014). If exploitation is too high, the spawning stock biomass and its realized reproductive output can decrease to the point that recruitment is impaired and declines with further reductions in the number of spawners (a condition known as recruitment overfishing), which may lead to the collapse of the fishery (Post et al., 2002). Aside from affecting population demography, size‐selective recreational fishing induces evolutionary changes of life history traits, including reductions in age and size at maturation and changes in growth rates (Dunlop, Shuter, & Dieckmann, 2007; Matsumura et al., 2011), life history tactics (e.g., anadromy vs. residency; Thériault et al., 2008), and behavioral traits (Sutter et al., 2012). Rates of phenotypic change in life history and behavioral traits induced by either commercial or recreational fishing strongly increase with the intensity of exploitation (Alós, Palmer, Trías, Díaz‐Gil, & Arlinghaus, 2015; Arlinghaus et al., 2009; Sharpe & Hendry, 2009).

Inland recreational fisheries are often managed via length‐based harvest regulations, although they often create trade‐offs among conservation and fishery goals (Gwinn et al., 2015). The most common regulation is a minimum‐length limit (MLL), where small, typically (and preferably) immature fish cannot be harvested and only fish over the MLL can be harvested. Harvest slot limit (HS) regulation implies a combination of minimum and maximum size limits, so that only fish of intermediate size are harvested. (See Gwinn et al., 2015 for a review of and conceptual background on length‐based harvest regulations.) Theoretical studies on commercially harvested stocks suggest that HS regulations would increase spawning stock biomass and yield while reversing the detrimental evolutionary effects induced by harvest and thus lead to higher evolutionary stable yield compared to classical harvest strategies based on MLL (Jørgensen, Ernande, & Fiksen, 2009; Zimmermann & Jørgensen, 2017). The scarce theoretical research (Arlinghaus, Matsumura, & Dieckmann, 2010; Matsumura et al., 2011) on the coupled eco‐evolutionary consequences of alternative recreational fisheries’ length‐based harvest regulations points to the same direction.

In this study, we added a fishing module to the eco‐genetic individual‐based model inSTREAM‐Gen (Ayllón et al., 2016) to explore the effects of harvest under different management strategies that use different types of length‐based harvest regulations (MLL vs. HSs) that vary in their restrictiveness and intensity of exploitation, on the eco‐evolutionary trajectory of the population and the sustainability of the recreational fish stock. We simulate a stream population of brown trout *Salmo trutta* L. for 100 years as our experimental system. We hypothesized that (1) size‐selective harvest through recreational fishing over 100 years would (a) decrease the spawning stock and overall abundance, and reduce the average size and age of the population, and (b) induce evolutionary responses on size at emergence and the maturity size threshold, with strong consequences on population dynamics; and (2) the effects would differ across regulation scenarios, being (a) stronger with increasing exploitation intensity and decreasing minimum‐length limit and (b) buffered by implementing a maximum‐length limit (MaxLL), the buffering effect increasing as the maximum harvestable length is decreased.

## 2 MATERIALS AND METHODS

### 2.1 Model description

InSTREAM‐Gen was implemented in the freely available software platform NetLogo 5.0.4 (Wilensky, 1999) and a detailed model description that follows the ODD (Overview, Design concepts, Details) protocol for describing individual‐based models (Grimm et al., 2006, 2010) is provided in Supporting Information Appendix S1. The model and its documentation are freely available online (https://github.com/DanielAyllon/inSTREAM-Gen-Fishing-version). The ecological structure of the model builds on inSTREAM (version 4.2; Railsback, Harvey, Jackson, & Lamberson, 2009); inSTREAM has been used since 1999 to address a wide range of applied and theoretical questions at over 40 rivers, and validation studies have shown that, under controlled conditions, it can predict individual‐ and population‐level effects of environmental change (e.g., Harvey, Nakamoto, White, & Railsback, 2014; Railsback & Harvey, 2002).

To this model, inSTREAM‐Gen added an inheritance model to allow for the genetic transmission of two fitness‐related traits that are independent of each other: size at emergence (the length of new fish produced in the model as they hatch from eggs) and maturity size threshold (minimum length for spawning), which is sex‐specific. Fishing‐induced evolutionary changes in maturity size threshold are well‐established (see Introduction). As our model implements a size‐based dominance hierarchy, larger size at emergence increases the probability of an offspring's survival and growth. As in real salmonid populations (Jonsson & Jonsson, 2011), the size‐based hierarchy means that an initial size advantage gives a newly hatched individual better access to safe and productive habitat. This positive feedback means that, while individual growth and survival depend on environmental conditions and behavior, size at emergence can have strong effects on lifetime fitness. Because size at emergence affects adult size, we hypothesize that fishing could affect selection for the size‐at‐emergence trait. We also included a heritable neutral trait (not affecting the fitness of individuals) to control for genetic drift.

In inSTREAM‐Gen, demographic and genetic dynamics emerge from the growth, survival, and reproduction of individuals, individual‐level processes that are driven by complex interactions among environmental conditions, habitat, competition for resources, and adaptive behavior. Therefore, plastic responses in growth rates, reproductive (e.g., size at first reproduction), and phenological (e.g., spawn timing) traits can emerge in inSTREAM‐Gen from natural and anthropogenic changes in the fish environment. Likewise, density‐dependent mortality and growth are emergent processes.

InSTREAM‐Gen is spatially explicit and describes one reach of a stream. The model simulates the complete trout life cycle using a daily time step, with stream flow and water temperature as the driving environmental variables. Spatial variation is represented via rectangular cells that represent patches of relatively uniform habitat and are characterized by both dynamic flow‐dependent (e.g., food production) and static (e.g., substrate and cover availability) variables. On each simulated day, environmental conditions in the reach and cells’ flow‐dependent variables are updated, and then trout individuals execute the following actions. (a) All trout select habitat following a size‐based dominance hierarchy by which larger trout get first access to food and preferred habitat. Each trout moves to the available cell that maximizes short‐term fitness, which is a function of the cell's mortality risk and growth potential (Railsback, Lamberson, Harvey, & Duffy, 1999). (b) Trout feed and grow according to their food intake and energy costs experienced in their cell, which are calculated through a bioenergetics model. (c) All trout are subject to six natural sources of mortality: high temperatures, high flow velocity, stranding, starvation, predation by terrestrial animals, and predation by piscivorous trout. These are modeled as daily survival probabilities that depend on characteristics of the fish and its habitat.

During the spawning (i.e., reproductive) season, mature females (individuals with body length over the phenotypic maturity size threshold) spawn if both environmental and internal conditions are met. If so, female trout create a redd and its eggs are fertilized by the largest available male spawner plus a random number of smaller mature males. The number of eggs increases exponentially with female length, and there is a trade‐off between egg size and number, because females have limited energy resources and limited body cavity space for egg production (Jonsson & Jonsson, 2011). In trout, egg size generally increases with spawner size and larger eggs produce larger offspring (Jonsson & Jonsson, 2011). To reproduce these patterns, egg size in our model increases with the genotypic value of the female's trait for size at emergence (see next point). Thus, females with a larger genetic value of size at emergence produce larger but fewer eggs than females with average genetic values. Each redd stores the genetic information of the mother and all contributing males.

Redds are modeled as individuals and are subject to egg mortality. Surviving eggs develop at a rate that increases with temperature. When eggs are fully developed, they hatch into new trout (emerge) and the heritable traits are transmitted.

Inheritance of size at emergence and maturity size are modeled by assuming each new trout inherits its genetic traits from the mother and one father randomly selected from the males that fertilized the redd, with equal probability of fertilization across males. We model the phenotype of an individual as the sum of an inherited additive genetic effect (genotypic value) and a nonheritable environmental effect, inheritance rules based on the infinitesimal model of quantitative genetics (Lynch & Walsh, 1998). The genotypic value for an evolving trait of each new trout is drawn from a normal distribution with mean equal to the arithmetic mean of the two parental values and variance equal to half the total additive genetic variance for the trait at the population level plus the variance potentially introduced by mutation. We assume that the additive genetic variance at the population level remains constant across generations.

### 2.2 Fishing module

The fishing mortality component follows the models implemented in inSTREAM‐SD (Railsback, Harvey, & Sheppard, 2013). The assumptions and technical implementation of the module are described in the Supporting Information Appendix S2 and parameter values discussed in Supporting Information Appendix S3. In summary, the fishing model includes three separate components: fishing pressure, capture rate, and survival. Fishing pressure is an input reflecting the intensity (person‐hours per day) of fishing in the reach and is assumed to be spatially homogeneous within the reach. Capture rate is the mean number of times a fish is captured per day and depends only on fishing pressure and fish length (capture being less likely for smaller fish). Survival depends on how many times a simulated trout is hooked, which is determined by a random draw from a Poisson distribution with capture rate as the mean, and whether it is kept vs. released each time hooked.

Whether a fish is kept or released depends on whether the fish is of legal size to harvest, which depends on the regulations being simulated. Under the MLL regulation, anglers can keep every caught fish larger than a fixed length threshold. Under the HS regulation, all fish smaller than a minimum‐length threshold and larger than a maximum‐length threshold are protected from harvest and must be released mandatorily; anglers can keep all fish with a size within the harvestable slot. In our modeled fishery, voluntarily catch‐and‐release fishing is allowed. Thus, in the model, whether a trout of legal size to harvest is finally kept is determined from a random draw from a uniform distribution defined by an input parameter. Therefore, the model assumes that a fraction (60% in this study; Supporting Information Table S3‐2 in Appendix S3) of the hooked fish of legal size are released by anglers. We implemented mortality from noncompliance with the harvest regulations by assuming that a fraction (5%) of the fish of nonlegal size was illegally kept by anglers. Trout can only be angled during the angling season (from April to October in this study).

Hooking mortality (i.e., the subsequent death of fish caught and released by anglers) is modeled as a separate, but related mortality source. Hooking mortality and illegal harvest are cryptic sources of mortality (Coggins, Catalano, Allen, Pine, & Walters, 2007) that can be important components of the impact of recreational fishing on fish populations and, thus, influence the effectiveness of harvest regulations (Johnston, Beardmore, & Arlinghaus, 2015). The model simply assumes that a fraction of released trout dies of hooking. The selected hooking mortality rate (20%; Supporting Information Appendix S3) is relatively high but within the ranges observed for the species, being a compromise between the average value (12.1 ± 6.7%) reported for brown trout angled with live bait (the one typically used in the modeled fishery), and the average value (27%) reported for salmonids angled with the same bait type (Hühn & Arlinghaus, 2011).

### 2.3 Simulation scenarios

#### 2.3.1 Baseline scenario

The model was parameterized to a brown trout fishery in northern Spain (Supporting Information Appendix S3). We modeled a baseline scenario that mimicked the observed variability in temperature and flow to simulate the dynamics of the population for a 1993–2100 time period. We used data collected by the closest meteorological and stream gauging stations to generate the water temperature and flow time series for 1993–2011. Time series for 2012–2100 were then projected following the methodology described in Ayllón et al. (2016). Under this baseline scenario, recreational fishing is not allowed so there is no mortality from either angling or hooking.

#### 2.3.2 Angling scenarios

To evaluate effects of fishing, we simulated the model population under 150 different angling scenarios. The angling scenarios are identical to the 1993–2100 baseline scenario except for using cross‐combinations of three angling parameters: exploitation rate (ExpR) and minimum and maximum‐length limits. The values of these parameters are described below:

##### Exploitation rate

ExpR is the percentage of the harvestable stock (i.e., trout that can be legally harvested) that is harvested (i.e., caught and kept by anglers). We simulated five levels of ExpR: 5%, 20%, 35%, 50%, and 65%. Model input for fishing pressure (expressed as angler‐hours per km and day) was calculated from ExpR as follows:

(1) $$\begin{array}{cc} \mathrm{anglePressure} & \;=\;(ExpR\;\times \;harvestableStock\\ & \;\times \;anglingE\;f\;f\;iciency)/(reachLength\;\times \;seasonLength) \end{array}$$

where *anglingEfficiency* is the number of angler‐hours necessary to catch and keep a trout, and *reachLength* and *seasonLength* are the length of the modeled reach (in km) and the angling season (in days); and *harvestableStock* is the number of trout that can be legally harvested during the angling season. The value of *harvestableStock* must be estimated at the beginning of the angling season, after which the fishing pressure is fixed for the entire season. The model estimates *harvestableStock* as the sum of the trout that have a legal size at the beginning of the angling season plus the trout that would reach legal size during the angling season. To estimate the latter, all trout project their growth over the whole season from the conditions experienced at the beginning of the season; if the projected size is greater than the minimum‐length limit, then the trout is considered within the harvestable stock. The mean deviation of the realized exploitation rate from the treatment level (due to the forward projection of *harvestableStock*) across angling scenarios was 12.0% (±1.9%, SE), while the median was 6.0%.

##### Minimum‐length limit

MinLL is the lower bound of the length range in which fish are legal to keep (cm). We used five levels: 17, 18, 19, 20, and 21 cm. (The actual minimum‐length limit at our study site has shifted between 19–20 cm over the last 25 years.) Comparing these values of MinLL to the actual length and age distributions observed at our site (see Supporting Information Appendix S3) lets us estimate how much of spawning population would be harvestable. Under the most restrictive regulation (MinLL = 21 cm), the harvestable stock at the beginning of the simulation consists only of age‐3 and older mature individuals (Table 1). Under the current regulation (MinLL = 19 cm), the harvestable stock also includes the largest 20% of age‐2 trout and a negligible proportion of immature fish (i.e., fish with a length below their phenotypic maturity threshold). Finally, if MinLL is reduced down to 17 cm, then almost 75% of age‐2 trout are of legal size, and 42% of the catchable age‐2 trout are immature.

**Table 1:** Percentage of individuals of each age class that are included in/excluded from the harvestable stock (trout that can be legally harvested) for each length‐limit scenario at the beginning of the simulation period (1993–2004). The percentage of the harvestable stock that is immature is in parentheses. No age‐1 trout are of legal size under any length‐limit scenario at the beginning of the simulation period

Length limit (cm)/Age class	Age‐2	Age‐3 and older
Minimum	Harvestable stock includes (%)	
17	72.6 (42.1)	100.0 (1.1)
18	45.2 (19.2)	100.0 (1.1)
19	20.1 (6.3)	100.0 (1.1)
20	5.8 (1.4)	100.0 (1.1)
21	0.0	88.7 (0.2)
Maximum	Harvestable stock excludes (%)	
25	0.0	28.4
27	0.0	19.3
29	0.0	11.9
31	0.0	6.7
33	0.0	2.8

##### Maximum‐length limit

This parameter is the upper bound of the legal length range (cm) when a HS regulation is implemented. Six levels were used: 25, 27, 29, 31, 33, and 100 cm. The 100‐cm level is equivalent to no upper length limit; hence, the performance of HS and MLL regulations can be compared. All of these values of MaxLL affect only age‐3 and older trout, the lowest one (25 cm) preventing almost one‐third of that age class from being legal to harvest (Table 1).

#### 2.3.3 Scenarios without evolution

As a side experiment, we simulated the effects of intensive fishing (ExpRs from 35% to 65%) under the least restrictive harvest regulation (MinLL = 17 cm, MaxLL = 100 cm) with no genetic transmission of traits. These scenarios were otherwise identical to those with evolution. Analysis of these scenarios allowed us to disentangle the purely phenotypic plastic responses (i.e., the case without evolution) from the one where both plastic and evolutionary responses happen (main analysis). Results are in Supporting Information Appendix S6.

### 2.4 Model outputs

We analyzed 15 population outputs that are recorded by the model each simulated year at September 1st. These outputs are density, biomass, and mean weight of four age classes (0, 1, 2, and 3 and older trout); the total population density and biomass; and the ratio of adult (age 2 and older) to juvenile (age 0 and 1) biomass in the population. We also recorded at the end of the spawning season the density and mean age of spawners; their mean sex‐specific genotypic maturity length threshold; the mean genotypic values of length at emergence and the neutral trait of all spawners; and the total number of eggs laid during the spawning season. The number of fish killed by angling and hooking was also recorded at the end of the angling season.

We executed six replicates (differing only in their random number sequence) of each scenario. (Measures of the variability among replicates of model outputs are included in Supporting Information Appendix S4).

### 2.5 Data analyses

We first tested whether values of each model output at the end of the simulation time frame (mean value over the last 15 simulated years, 2086–2100) under each fishing scenario significantly differed from those under the baseline using a pairwise *t* test with alpha equal to 0.05. Second, we applied the nonparametric Mann–Kendall trend test to determine whether there was a significant upward or downward trend over time in model outputs compared to the baseline scenario (see Supporting Information Appendix S5). Third, we assessed the effect of each angling parameter (ExpR, MinLL, and MaxLL) on the mean value over the last 15 simulated years (2086–2100) of model eco‐evolutionary outputs. To do this, we performed factorial ANOVAs, including the three angling parameters as independent factors and accounting also for their interaction (150 combinations overall), and analyzed both the direction of the effect and its magnitude. For the latter, we decomposed the percentage of variance explained by each angling parameter using the *relaimpo* package v2.2‐2 for R (Groemping & Matthias, 2015). All statistical analyses were performed with the R software v. 3.3.3 (R Core Team 2017).

## 3 RESULTS

### 3.1 Statistical comparison with the baseline scenario

**Figure 1:**
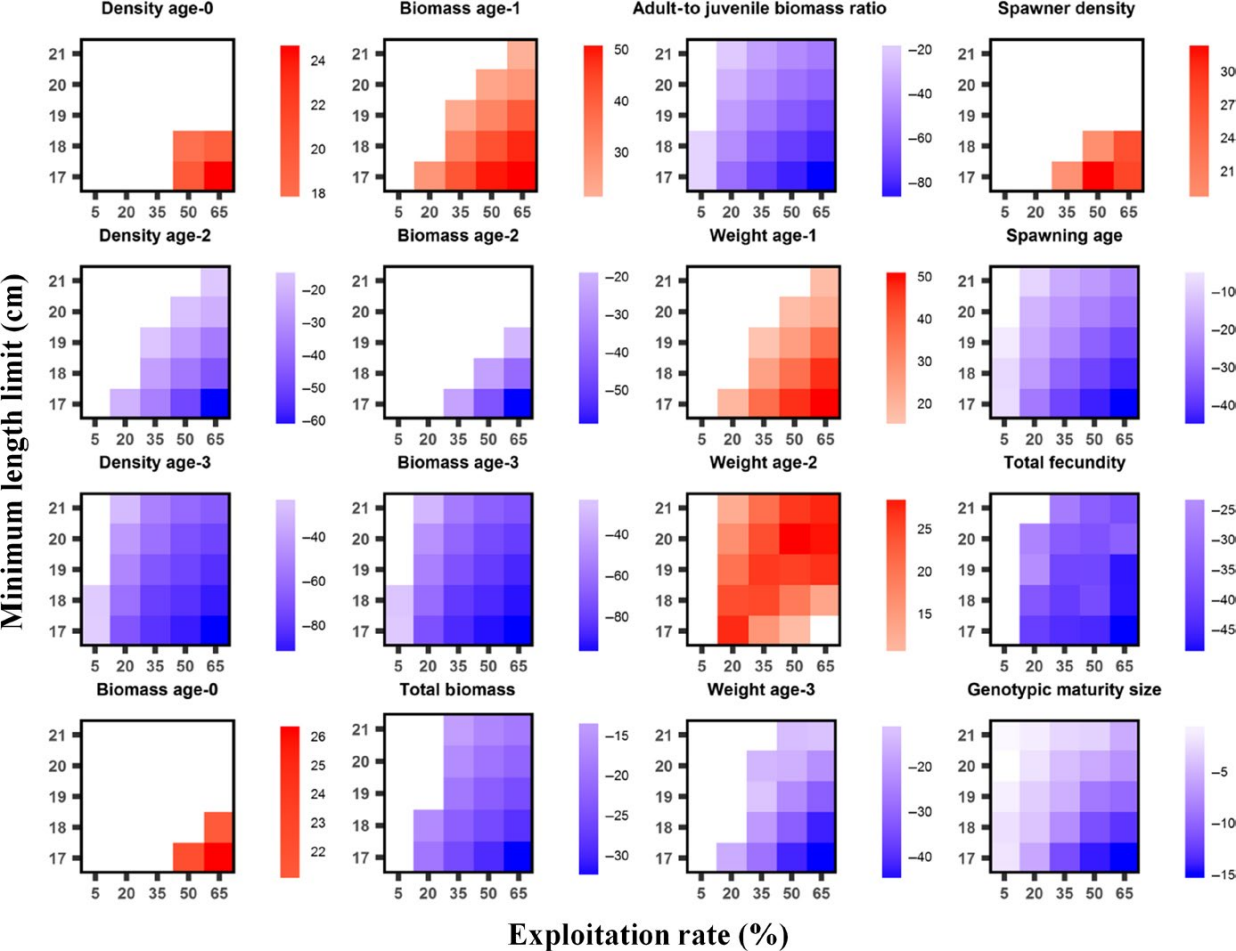
Effect of exploitation rate and minimum‐length limit on population eco‐evolutionary outputs at final simulation time for maximum‐length limit of 100 cm (i.e., no slot‐length limits and only minimum‐length limit). For each graph, a white color indicates that the simulation results under the angling scenario are not significantly different (pairwise *t* tests, *p *<* *0.05) from the baseline scenario of no angling. Blue/red shades indicate the strength of decrease/increase in the angling scenario compared to the baseline. Color scales on the right of each graph indicate the ranges of significantly different mean values over the last 15 simulated years (2086–2100) of six simulation replicates expressed as the percentage change: [(mean scenario − mean baseline)/mean baseline] × 100. Total density, density of age‐1 trout, weight of age‐0 trout, genotypic length at emergence, and neutral trait values of spawners did not significantly differ from the baseline scenario under any angling scenario so are not graphed

We did not detect significant changes from the baseline in total population density and density of age‐1 trout under any angling scenario. Density and biomass of age‐0 trout and number of spawners only significantly increased in relation to the baseline under the most intensive angling scenarios (highest ExpRs and smallest MinLLs; Figure 1). The rest of population traits significantly differed from the baseline under most angling scenarios (Figure 1). Regarding genetic traits, the genotypic value of minimum length for spawning was significantly lower than the baseline scenario value in almost all angling scenarios (Figure 1), but no changes in genotypic values of length at emergence and neutral trait were detected under any angling scenario.

**Figure 2:**
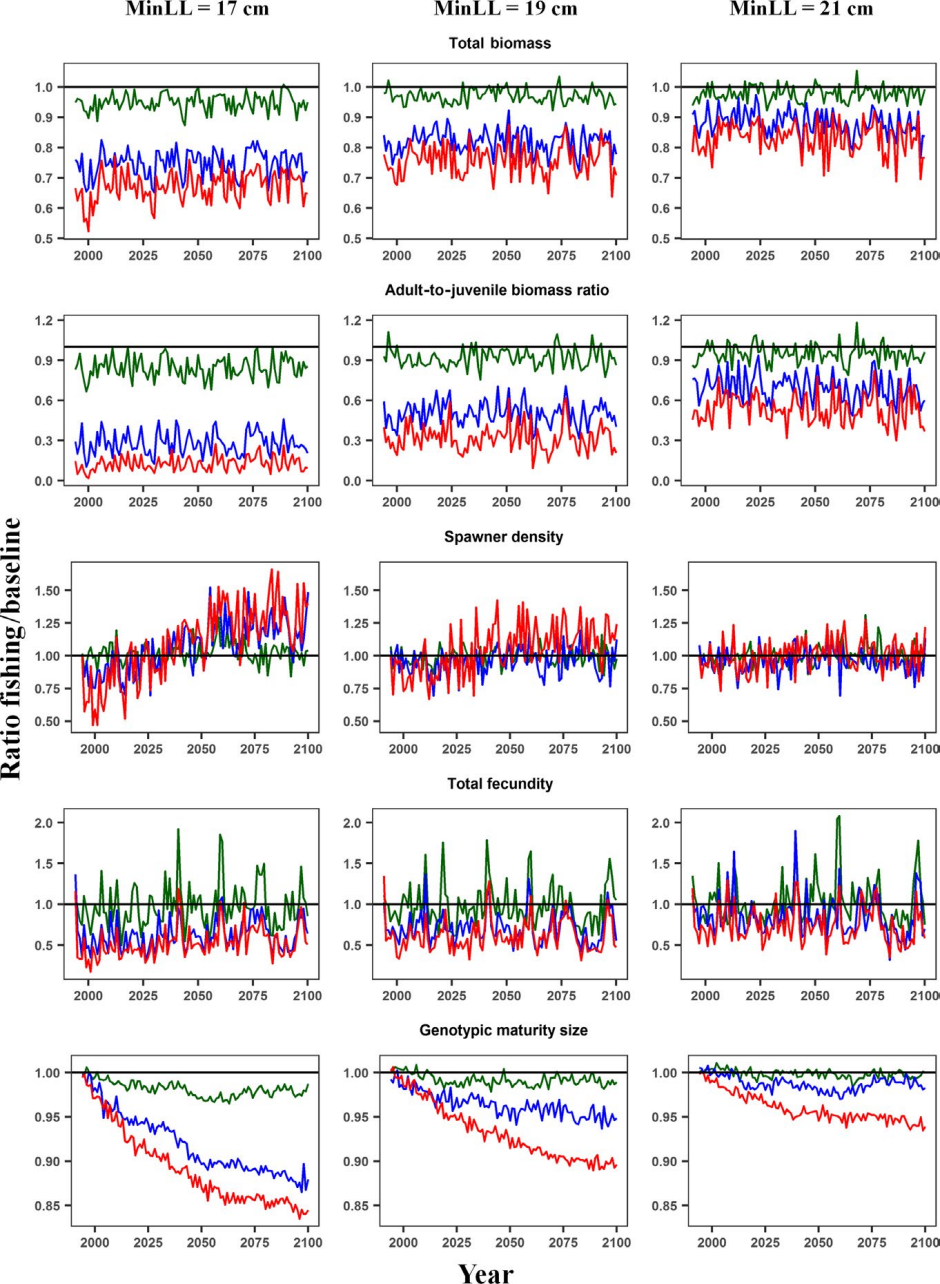
Ratio of five model outputs under different angling scenarios to the baseline scenario (no angling) over time. The angling scenarios are combinations of three levels of minimum‐length limit (17, 19, and 21 cm) and exploitation rate (5, 35, and 65%, plotted as green, blue, and red lines, respectively). The maximum‐length limit was set to 100 cm (i.e., no slot‐length limits). Trajectories of model outputs are averages of six replicates

Harvest quickly reduced total biomass and the ratio of adult to juvenile biomass (the population was truncated) at the start of simulations, but there were no significant trends in those outputs over time under most fishing scenarios (Figure 2 and Supporting Information Figure S5‐2). In contrast, there was a significant decrease in age‐2 and older trout body size over time in scenarios with higher ExpR and lower MinLL, while body size of age‐1 trout experienced the opposite trend (Supporting Information Figures S5‐1 and 2). The genotypic value of minimum length for spawning showed also a significant downward trend, and consequently, the number of spawners increased over time after the strong decrease in the first years of simulation (Figure 2). These trends were stronger with increasing ExpR and decreasing MinLL. Despite the decrease in size of age‐2 and older trout under the scenarios of intense exploitation and little restriction on harvestable length, total fecundity increased over time due to the positive trend in the number of spawners (Figure 2 and Supporting Information Figure S5‐2).

### 3.2 Effects of exploitation rate

ExpR exerted stronger effects than length‐limit parameters on most population outputs (Table 2). Increasing ExpR had a positive effect on density and biomass of age‐0 and biomass of age‐1 trout but negative effect on older trout density and biomass, so total density slightly increased, while total biomass strongly decreased (Table 2, Figure 1), and thus, the population was truncated. While the mean individual weight of age‐0 and age‐1 trout increased with increasing ExpR, the mean weight of age‐3 and older trout decreased. Regarding the genetic and reproductive traits, ExpR did not exert a significant effect on the genotypic value of length at emergence; in contrast, the genotypic value of minimum length for spawning, and thus, the mean age at which trout spawned decreased with increasing ExpR. As a consequence of all these results, the number of spawners slightly increased, while the total number of eggs produced by them strongly decreased with increasing ExpR. Increasing ExpR increased the total number of trout killed by hooking (Table 2).

**Table 2:** Effects of angling parameters (minimum‐ and maximum‐length limits and exploitation rate) and their interactions on population eco‐evolutionary outputs. Symbols show the direction (+ increase vs. − decrease) and significance of the effect (n.s. nonsignificant, *p <* *0.05, **p <* *0.01, ***p <* *0.001) as well as its magnitude (percentage of variance explained)

Population responses	MaxLL	MinLL	ExpR	MaxLL: ExpR	MinLL: ExpR
Density total (trout/ha)	(+)* [0.8]	(−)*** [24.4]	(+)*** [69.0]	n.s.	*** [4.2]
Density age‐0	(+)* [0.2]	(−)*** [24.8]	(+)*** [68.7]	n.s.	*** [6.0]
Density age‐1	(+)* [2.9]	(−)*** [5.8]	(+)*** [45.7]	n.s.	*** [39.6]
Density age‐2	(+)* [0.2]	(+)*** [33.2]	(−)*** [54.0]	n.s.	*** [12.6]
Density age‐3Plus	(−)*** [0.3]	(+)*** [15.1]	(−)*** [83.1]	* [0.1]	*** [1.3]
Weight age‐0 (g)	n.s.	(−)*** [30.3]	(+)*** [21.7]	n.s.	** [31.7]
Weight age‐1	(+)*** [0.2]	(−)*** [30.2]	(+)*** [58.7]	n.s.	*** [10.9]
Weight age‐2	(+)*** [0.4]	(+)*** [6.4]	(−)*** [58.3]	n.s.	*** [33.9]
Weight age‐3Plus	(−)*** [2.6]	(+)*** [28.4]	(−)*** [51.5]	n.s.	*** [15.7]
Biomass total (kg/ha)	(−)*** [0.3]	(+)*** [20.0]	(−)*** [76.8]	n.s.	*** [2.7]
Biomass age‐0	(+)** [0.1]	(−)*** [27.1]	(+)*** [64.4]	n.s.	*** [8.2]
Biomass age‐1	(+)*** [0.2]	(−)*** [28.9]	(+)*** [63.1]	n.s.	*** [7.6]
Biomass age‐2	(+)** [0.2]	(+)*** [41.1]	(−)*** [30.3]	** [0.2]	*** [28.2]
Biomass age‐3Plus	(−)*** [0.6]	(+)*** [14.2]	(−)*** [83.5]	** [0.2]	*** [1.5]
Ratio adults to juveniles (unitless)	(−)*** [0.3]	(+)*** [21.9]	(−)*** [74.7]	** [0.1]	*** [3.0]
Density spawners (trout/ha)	n.s.	(−)*** [34.4]	(+)*** [34.9]	n.s.	*** [28.3]
Number eggs (eggs/ha)	n.s.	(+)*** [13.9]	(−)*** [81.4]	n.s.	* [2.7]
Spawning age male (days)	(−)*** [0.4]	(+)*** [23.2]	(−)*** [71.9]	n.s.	*** [4.2]
Spawning age female	(−)*** [0.4]	(+)*** [23.3]	(−)*** [71.9]	n.s.	*** [4.1]
Gen min spawn length male (cm)	(−)** [0.2]	(+)*** [32.2]	(‐)*** [57.2]	n.s.	*** [9.9]
Gen min spawn length female	(−)** [0.2]	(+)*** [32.5]	(−)*** [57.1]	n.s.	*** [9.8]
Gen emergence length	n.s.	n.s.	n.s.	n.s.	n.s.
Gen neutral trait (unitless)	n.s.	n.s.	n.s.	n.s.	n.s.
Hooked dead fish (trout/ha)	n.s.	(−)*** [17.3]	(+)*** [77.0]	n.s.	*** [5.5]

Note

+ The interaction term Max Length:Min Length was nonsignificant for all model outputs.

### 3.3 Effects of length limits

The effect of the MinLL on all population outputs was opposite that of ExpR (Table 2, Figure 1): decreasing the MinLL increased the numbers and size of age‐0 and age‐1 trout, so the total population density increased, while the numbers of age‐2 and older trout decreased. The total population biomass and ratio of adult to juvenile biomass also decreased. The genotypic value of minimum length for spawning evolved to lower values when we reduced MinLL, so the mean age at spawning and the total number of eggs produced also decreased even though the number of spawners increased. Decreasing the MinLL increased the total number of trout killed by hooking (Table 2).

Implementing a MaxLL had little effect on most population outputs (Table 2). Increasing the MaxLL (i.e., decreasing the proportion of the largest fish that are protected from harvest) increased the density and biomass of age‐0, age‐1, and age‐2 trout, and thus total population density, and the mean weight of age‐1 and age‐2 trout. In contrast, increasing MaxLL had a negative effect on density, mean weight, and thus biomass, of age‐3 and older trout, which resulted in lower total population biomass and lower ratio of adult to juvenile biomass. Decreasing the number of the oldest fish consequently reduced the mean age at spawning. Increasing MaxLL had a weak negative effect on the genotypic value of minimum length for spawning but no effects on the length at emergence, nor on the number of fish killed by hooking.

### 3.4 Interactive effects of exploitation rate and length limits

There were no significant interactions between MaxLL and MinLL for any population output (Table 2). The interaction of MaxLL and ExpR was significant only for four model outputs, but the effects were weak (Table 2). In contrast, we detected significant interactive effects of MinLL and ExpR on all population outputs (Table 2), but only in a few cases were the effects really strong. In general, the interaction of both factors reinforced their separate effects (i.e., their effects were synergistic). As a result, population responses were strongest under the most aggressive fishing scenarios (high ExpR and small MinLL), and only under these scenarios was the number of spawners significantly higher than in the baseline scenario (Figure 1). There were only two exceptions to this pattern (Figure 1 and Supporting Information Figure S5‐2): mean weight of age‐2 trout, which increased with increasing ExpR when the MinLL is large (more age‐3 and adult trout are harvested), but decreased with increasing ExpR when the MinLL is small (more age‐2 trout are harvested); and density of age‐1 trout, for which the effect of MinLL was positive at low ExpRs but negative at the highest ExpRs, having a nonlinear relationship.

## 4 DISCUSSION

Harvest‐induced adaptive changes in life history and physiological and behavioral traits are ubiquitous in commercially and recreationally exploited fish stocks. Management of exploited stocks, and especially the recovery of those overexploited, is challenging when such phenotypic changes result at least in part from evolutionary changes. Predictive mechanistic models that include relevant environmental drivers and structures and are based on first principles such as bioenergetics, fitness seeking and natural selection can support decision‐making in light of the expected consequences of alternative harvest regimes on eco‐evolutionary trajectories over time frames that are relevant for management.

Our simulation results supported our hypothesis that increasing Exploitation rate (ExpR) or decreasing minimum‐length limit (MinLL) would lead to reduced average size and age of individuals and depressed spawning stock biomass and reproductive output. Main findings from our simulations were: (a) fishing triggered evolutionary and plastic responses leading to changes in maturation schedules and growth trajectories that compensated for the truncation of age and size distributions within the population and prevented its collapse even under intense fishing pressure and less restrictive harvest regulations; (b) the rapid evolution of the size maturity threshold was necessary for such compensation, because density‐dependent growth and growth‐mediated plasticity of sexual maturity alone could not prevent recruitment overfishing; (c) high rates of cryptic mortality amplified the demographic consequences of harvest and might alter the evolutionary trajectory of individual growth; and (d) the implementation of a maximum‐length limit (MaxLL) on fishing had positive effects on population abundance and structure, but its ability to buffer fishing‐induced demographic impacts was hindered by cryptic mortality sources.

### 4.1 Population traits and evolutionary responses

In our simulations, increasing exploitation intensity and decreasing MinLL increase selection for earlier maturation at small size. This pattern is in line with previous empirical and theoretical research on the responses of maturation schedules in harvested marine (e.g., Dunlop et al., 2009; Piou et al., 2015) and freshwater fish populations (e.g., Edeline et al., 2007; Matsumura et al., 2011; Wang & Höök, 2009). We observed the strongest evolutionary responses at high fishing ExpRs and small MinLLs, when the number of harvested immature individuals increased, and thus, selective pressures favored those individuals that matured prior to being harvested. As a result of this earlier maturation, the number of simulated spawners actually increased under such regulations, because the proportion of age‐1 and age‐2 trout that became spawners increased sharply. While the total number of eggs produced was considerably reduced because smaller spawners are less fecund, the fishery‐induced evolutionary responses still kept the spawning potential ratio (spawning stock biomass per recruit (SSBR) of a fished stock divided by the SSBR of the stock before it was fished) above the values that indicate the potential for recruitment overfishing (0.35; Arlinghaus et al., 2010). However, such a large fraction of spawners that are young can lead to unstable population dynamics characterized by nonlinear amplification of environmental stochasticity by biological processes (Anderson et al., 2008).

The simulated population eco‐evolutionary time trajectories indicated that, after 100 years of harvest, plastic and evolutionary compensatory changes in maturation schedules could buffer the demographic impacts of fishing, even under intense fishing pressure and less restrictive harvest regulations. However, the modeled population became highly unstructured in the first 25 years following the implementation of the permissive regulations; the number and size of simulated spawners, and thus total fecundity, sharply decreased to levels that would expose the population to demographic collapse induced by stochastic environmental events. The higher the ExpR and the smaller the MinLL, the longer it took for the maturity threshold trait to stabilize, and the trait was still evolving after 100 years of fishing under the simulated scenarios with the highest exploitation intensities and least restrictive regulations.

In contrast to the maturity size threshold, our simulations did not show evolutionary changes in the size‐at‐emergence trait. In our model, individual dominance, access to food, and therefore growth increase with size at emergence; therefore, we expected phenotypes with larger size at emergence to be selected under high harvest intensity and a small MinLL close to the maturity size, as individuals growing faster would have higher chances of experiencing at least one successful spawning event. In general, acceleration of life history would be favored when the associated mortality risk is lower than the extra mortality accumulated by a slower life history (Enberg et al., 2012). Since when MinLL is large compared to the maturity size fishing mostly targets mature individuals, individuals staying longer below the MinLL would have more spawning opportunities, and so natural selection would select for slow growers, as found in previous studies (e.g., Dunlop et al., 2009; Matsumura et al., 2011). However, in our model capture probability increases with fish size up to about 25 cm, so under scenarios with intense exploitation and small MinLL, larger individuals face higher mortality risk because their size is legal to harvest for more of the angling season (April–October) before the spawning season starts (November). At the same time, slow growers may not attain the maturity size threshold by age‐2 and thus would have to survive another whole angling season before spawning. Therefore, there would be selection for mature individuals that reach MinLL as late as possible during the angling season (average growers). These results reinforce the notion that changes in life history traits in response to fishing depend on the species ecology and local harvesting patterns and thus are difficult to predict (Enberg et al., 2012; Uusi‐Heikkilä et al., 2015).

### 4.2 Evolutionary versus plastic responses

In our model, intensive fishing of the largest individuals resulted in strong density‐dependent increases in size‐at‐age and survival of age‐0 and age‐1 trout mainly due to decreased competition for food and habitat. Changes in individual growth trajectories in conjunction with evolutionary change in the maturity size threshold led to alterations in the maturation schedules and reproductive patterns of the modeled stock, which buffered the harvest impacts on total fecundity. In fact, our results illustrate that fishing‐induced evolutionary responses are critical to buffer the demographic impacts of intensive harvest, as shown by the experiment in which we simulated the effects of intensive fishing and less restrictive length limits in a hypothetical nonevolving population (Supporting Information Appendix S5). The biomass of age‐2 and older trout, the ratio of adult to juvenile biomass, the number of spawners, and total fecundity were markedly lower without compared to with evolution (Supporting Information Table S6‐1). Without evolution, the spawning potential ratio declined to values that indicate overfishing (0.24). Therefore, evolutionary changes in life history traits not only led to a more natural and stable population structure but also decreased the risk of recruitment overfishing. This result indicates that, if evolutionary changes are not rapid enough, or there are other selective pressures operating in opposite direction, the population may decline rapidly due to overfishing, as often reported for wild stocks (e.g., Almodóvar & Nicola, 2004; Post et al., 2002).

### 4.3 Cryptic mortality and its consequences

The intercohort density‐dependent increase in numbers and size at age in simulated age‐1 trout under the fishing scenarios with the highest exploitation intensities and least restrictive regulations implied higher capture rates. An important side effect of higher capture rates of individuals below MinLL was the substantial increase in the number of simulated immature fish killed by hooking. Therefore, hooking mortality might exert a selective pressure that opposes the evolutionary response to size‐selective harvest mortality, counterselecting for slower growth. This might explain why we did not detect selection for larger size at emergence under such simulation scenarios. In addition, the number of simulated trout of legal size killed by hooking amounted to as high as the 50% of the total legally harvested trout. It follows that under these simulation scenarios, the number of individuals killed by hooking was even higher than total number of harvested trout, leading to very low harvesting efficiencies (i.e., proportion of fishery‐related biomass losses attributable to harvest).

### 4.4 Minimum versus harvest‐slot length limits

The implementation of a harvest slot regulation (HS) via maximum‐length limit (MaxLL) has significant positive but weaker than expected effects on population eco‐evolutionary dynamics. This was surprising, as previous modeling studies have shown that HS length limits change fishing‐induced selection pressures on maturation size and growth capacity (Matsumura et al., 2011) and increase harvest biomass and reduce truncation of the population's age structure compared to MinLLs in pike (Arlinghaus et al., 2010; Matsumura et al., 2011). Likewise, Gwinn et al. (2015) found that HSs increased the total number of fish harvested while reducing size truncation compared to MLLs across a range of exploitation intensities, fish life history strategies, and fisheries objectives; however, the benefits of HS regulations in their study were undermined by lowered biomass yields and smaller size of fish harvested.

There are three potential explanations for our results. (a) The tested MaxLLs are too large to induce strong buffering effects on the population eco‐evolutionary dynamics, as the most restrictive scenarios freed between 20 and 30% of the largest trout from being harvested. (b) InSTREAM‐Gen does not incorporate size‐dependent maternal effects on egg size, which in real populations affects offspring's performance traits such as growth and survival (Hixon et al., 2014), and thus, some important mechanisms through which very large individuals foster long‐term population stability were not accounted for. Nevertheless, given that in our simulations fishing did not select for larger size at emergence, size‐dependent maternal effects on egg size are unlikely to explain our observed patterns. (c) Under the highest exploitation intensities, the reduction in the number of large trout harvested is counterbalanced by higher mortality due to hooking (up to 75% higher under the scenario with the highest exploitation rate), as most of the protected fish died after repeated catch and release. It should be noted that the hooking mortality rate used in this study (20%) is relatively high and a lower hooking mortality rate should make harvest slot limits more effective. Our result adds evidence to previous work (e.g., Gwinn et al., 2015) showing that high rates of hooking mortality might render HS regulations ineffective at sustaining breeder biomass and total fecundity under high exploitation intensity. The effectiveness of the harvest regulation to mitigate demographic impacts and manage fishing‐induced evolution thus depends on the sensitivity of the fished species to hooking. Hence, in the same way as in commercial fisheries, discard rates and bycatch are considered fishing‐related externalities that should be incorporated into quantitative analyses to detect and avoid unsustainable harvest regimes (Laugen et al., 2014), hooking mortality must not be ignored when setting optimal regulations in a recreational fishery.

### 4.5 Management implications

**Figure 3:**
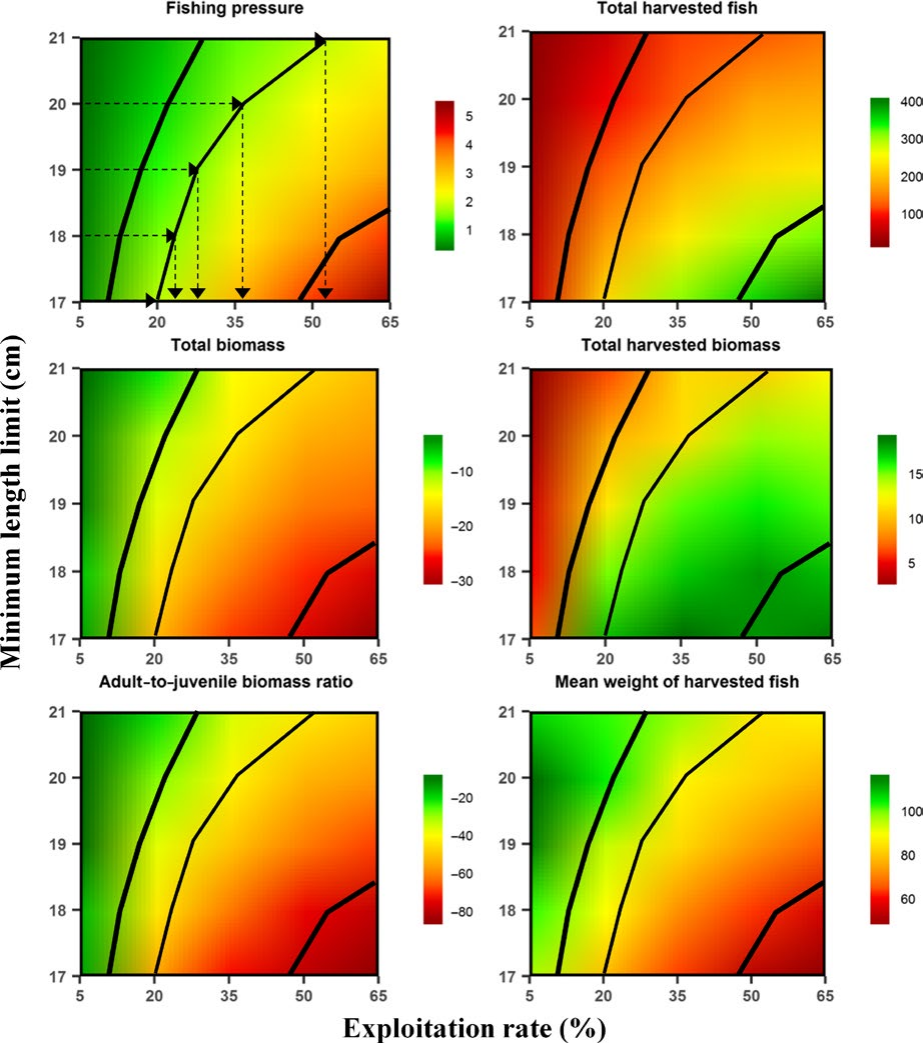
Example application of the study findings to fishery management. A target fishing pressure (expressed as angler‐h km^−1^ day^−1^) defines the estimated number of trout that will be harvested over the angling season (product of exploitation rate and size of harvestable stock in equation (1)). This estimated harvest can be reached through different combinations of exploitation rate and minimum‐length limit (top‐left plot). In the example, the minimum‐length limit defines the harvestable stock and thus the maximum exploitation rate (dashed lines and arrows). The different harvest regulations result in different demographic and fishery outputs. Demographic outputs are total biomass (kg/ha; mid‐left plot) and the ratio of adult to juvenile biomass (unitless; bottom‐left plot), expressed as the percentage change from the baseline scenario: [(mean scenario − mean baseline)/mean baseline] × 100. Fishery outputs include total harvested trout (fish/ha; top‐right plot), total harvested biomass (kg/ha; mid‐right plot), and mean weight of harvested trout (g; bottom‐right plot). The response surfaces for model outputs were obtained through linear contour interpolation. The central thin black line is the isoline for a target fishing pressure of 2 angler‐h km^−1^ day^−1^, while the thick lines to the left and right are the isolines for half and double fishing pressure (1 and 4 angler‐h km^−1^ day^−1^, respectively)

Our simulation results show that the emergent, intertwined ecological and evolutionary responses to harvest can be complex and sometimes counterintuitive. The recreational fishery manager should always consider sociological, biological, and ecological aspects in designing regulations that best balance conservation (e.g., maintaining natural age structure or spawner biomass) and fishery objectives (e.g., maximizing harvest yield or trophy‐size catches). As an illustrative example, a target fishing pressure, which sets the number of angling licenses, defines the potential harvest yield, which could be realized in our modeled fishery by means of different regulations, that is, different combinations of ExpR (e.g., by imposing daily bag limits) and MinLL (Figure 3; here, setting the MinLL defines the maximum ExpR that can be applied to the harvestable stock to meet the estimated harvest yield). However, these alternative ways of reaching the same yield have the following different effects on the eco‐evolutionary trajectories of the simulated harvested population.

Under an intermediate fishing pressure (Figure as above), both numbers and biomass of harvested fish are maximized by decreasing MinLL, but intermediate fishing pressure also produces a stronger truncation in the size distribution of the population and a reduction in the mean size of the harvested fish, as the proportion of age‐2 fish in the harvest markedly increases. On the other hand, for a fixed fishing pressure, increasing MinLL drives up hooking mortality of age‐1 and age‐2 fish (capture probability increases due to positive density‐dependent effects on growth). High rates of hooking mortality decrease harvesting efficiency but this effect is reduced when MinLL is smaller because more fish are harvested instead of released. Thus, in our modeled fishery, the best compromise between harvest efficiency and yield, and conservation is attained at an intermediate MinLL of 19 cm. Reducing the fishing pressure increases the optimal MinLL because larger limits result both in a more natural population structure (i.e., closer to historical prefishing values) and larger size of harvested trout while maintaining high yield. Finally, a very high target fishing pressure can only be attained by decreasing the MinLL down to 17–18 cm and increasing ExpR to very high levels (>50%), which would put at risk the population's persistence and the fishery's sustainability. Such high fishing pressure would lead to a young and presumably very unstable population, and the increased harvest biomass would consist of younger and smaller trout, which would decrease the perceived quality of the fishery by anglers (Johnston et al., 2015). Therefore, if overexploited recreational or commercial fish stocks are to be recovered, management actions must simultaneously target at reducing both fishing effort and hooking/bycatch mortality; once cryptic mortality rates are under control, HSs regulations should lead to improved sustainability and increased yield (e.g., Arlinghaus et al., 2010; Zimmermann & Jørgensen, 2017).

While this modeling exercise shows the complex population and yield patterns emerging from different harvest regulations, it is still a simple example. We did not account for potential changes in fish catchability resulting from inverse density dependence (e.g., Hunt, Arlinghaus, Lester, & Kushneriuk, 2011) or behavioral adaptation (e.g., Alós et al., 2015), nor angler behavior and its dynamics (e.g., Johnston et al., 2015). There is an increasing consensus that recreational fisheries must be approached from a systems perspective and conceptualized as complex adaptive social–ecological systems with multiple interactions and feedbacks among their social and ecological layers across multiple spatial scales (Arlinghaus et al., 2017). Yet, studies focused on isolated fisheries help to understand how alternative harvest regulations—even though the underlying social generative mechanisms are not explicitly accounted for—shape the demographic and evolutionary dynamics of the exploited stock and affect its sustainability.

## CONFLICT OF INTEREST

None declared.

## AUTHOR CONTRIBUTIONS

DA, SR, SV, and VG conceived and developed the model. DA, AA, GGN, and VG designed the experiment. DA, AA, GGN, and BE collected essential field data. DA performed the experiment and analyzed the data. DA wrote the first draft. All authors significantly contributed to the final manuscript.

## DATA ACCESSIBILITY

The model, its documentation, and input data are available at https://github.com/DanielAyllon/inSTREAM-Gen-Fishing-version.
