# Supplementary material for: Eco‐evolutionary responses to recreational fishing under different harvest regulations
Source: Ecol Evol. 2018 Sep 11;8(19):9600–13. doi: 10.1002/ece3.4270 (PMC6202708; doi:10.1002/ece3.4270)
Supplement: Supplementary file 1 [file ECE3-8-9600-s001.docx]

**Appendix S1:**

# Model description

Index:

[1.1 Purpose 1](#_Toc412759132)

[1.2 Entities, state variables, and scales 1](#_Toc412759133)

[1.3 Process overview and scheduling 3](#_Toc412759134)

[1.4 Design concepts 4](#_Toc412759135)

[1.5 Initialization 6](#_Toc412759136)

[1.6 Input data 7](#_Toc412759137)

[1.7 Submodels 7](#_Toc412759138)

## 1.1 Purpose

InSTREAM-Gen was designed to understand how environmental conditions and anthropogenic disturbances drive the evolution of demographics and life-history strategies of stream-dwelling trout populations. Therefore, it is particularly suited to simulate the eco-evolutionary consequences of river management decisions under a climate change context.

## 1.2 Entities, state variables, and scales

*Spatial scales*: The entire model is represented by one spatially-explicit stream reach of a length defined by the user, but never longer than 300 meters nor wider than 50 meters. The stream habitat within the reach is depicted as a grid of cells of variable size.

*Temporal scale*: The model includes a temporal scaling factor which allows the user to set the time step (never less than one day), so that it is user-specified. At any case, there are three trout actions (habitat selection, feeding and growth, and survival) which are always performed on a daily basis irrespective of the time step defined. The extent (duration) of the simulation is also defined by the user through the length of the environmental and habitat time-series.

*Entities*: This IBM includes three types of entities: Cells, trout and redds. Cells are objects that represent patches of relatively uniform habitat within a reach. Trout are modelled as individuals. Redds are spawning nests made by trout that are modelled as individual objects.

*State variables*: The global (reach) environment is characterized by its environmental and biological conditions. Each cell is characterized both by its physical habitat, and also by its production rate of two different kinds of food, drift and search (stationary) food. Each trout has 21 state variables, while redds’ state is described through 18 variables (Table S1-1).

**Table S1-1.** Agents included in inSTREAM-Gen with their state variables and units of measurement.

| **Agent** | **Variable** | **Description** | **Unit** |
| --- | --- | --- | --- |
| Cells | cellArea* | Area of the cell | cm^2^ |
|  | CellAreaCover* | Area of the cell with cover | cm^2^ |
|  | cellAreaShelter* | Area of the cell with velocity shelters | cm^2^ |
|  | cellDepth | Value of depth at specific time | cm |
|  | cellDistanceToHide | Average distance from hiding cover from the cell’s center | cm |
|  | cellFracCover* | Fraction of the cell with cover | Unitless (0-1) |
|  | cellFracGravel* | Fraction of the cell with spawning gravel | Unitless (0-1) |
|  | cellFracShelter* | Fraction of the cell with velocity shelters | Unitless (0-1) |
|  | cellFracSpawn* | Fraction of the cell with spawning gravel | Unitless (0-1) |
|  | cellNumber* | Number of the cell | number |
|  | cellVelocity | Value of velocity at specific time | cm s^-1^ |
|  | driftHourlyCellTotal | Production rate of drift food items | g h^-1^ |
|  | my-adjacentCells* | Adjacent cells | agents |
|  | my-patches* | Patches composing the cell | agents |
|  | searchHourlyCellTotal | Production rate of search food items | g h^-1^ |
|  | Transect* | Transect where the cell is located | number |
| Redds | creationDate* | Date when the redd is created | date |
|  | days-after-hatch | Number of days since emergence starts in the redd | days |
|  | eggsLostToDewateringTot | Total number of eggs lost due to scouring | eggs |
|  | eggsLostToHighTempTot | Total number of eggs lost due to high water temperatures | eggs |
|  | eggsLostToLowTempTot | Total number of eggs lost due to low water temperatures | eggs |
|  | eggsLostToScourTot | Total number of eggs lost due to dewatering | eggs |
|  | eggsLostToSuperimpTot | Total number of eggs lost due to superimposition of redds | eggs |
|  | fracDeveloped | Developmental status of a redd’s eggs | Unitless (0-1) |
|  | my-cell* | Cell where the redd is located | cell-id |
|  | numberOfEggs | Number of eggs in the redd | eggs |
|  | numberOfHatchedEggs | Number of eggs hatched (creating a new trout) | eggs |
|  | reddFathersgenNeutralTrait* | Genotypic values of neutral trait of fathers | User-specific |
|  | reddFathersgenNewlength* | Genotypic length at emergence of fathers | cm |
|  | reddFathersgenSpawnMinLength* | Genotypic minimum length to spawn of fathers | cm |
|  | ReddID * | Identity number of the redd | id |
|  | reddMothergenNeutralTrait* | Genotypic value of neutral trait of the mother | User-specific |
|  | reddMothergenNewlength* | Genotypic length at emergence of the mother | cm |
|  | reddMothergenSpawnMinLength* | Genotypic minimum length to spawn of the mother | cm |
| Trout | age | Number of days since the fish was born | days |
|  | age-class | Age class | Age0-Age5Plus |
|  | CauseOfDeath* | Mortality source by which the fish is dead | 8 sources |
|  | cMax | Physiological maximum daily intake | g d^-1^ |
|  | energyAvailableforGrowth | Net energy gain in my-cell during the time step | J d^-1^ |
|  | fishCondition | Condition factor | Unitless (0-1) |
|  | fishLength | Body length | cm |
|  | fishMaxSwimSpeed | Maximum sustainable swimming speed | cm s^-1^ |
|  | fishNeutralTrait* | Phenotypic value of the neutral trait | User-specific |
|  | fishNewLength* | Phenotypic length at emergence | cm |
|  | fishSpawnMinLength* | Minimum length to spawn | cm |
|  | fishWeight | Body weight | g |
|  | genNeutralTrait* | Genotypic value of the neutral trait | User-specific |
|  | genNewLength* | Genotypic length at emergence | cm |
|  | genSpawnMinLength* | Genotypic minimum length to spawn | cm |
|  | is-sheltered? | Access to a velocity shelter | True/False |
|  | maturityStatus | Maturity status | mature/non-mature |
|  | my-cell | Cell where the fish is located | cell-id |
|  | sex* | Sex | M/F |
|  | spawnedThisSeason? | Spawned this spawning season | true/false |
|  | status | Status | alive/dead |

* Fixed state variables.

## 1.3 Process overview and scheduling

*Processes*: The model is developed to cover the whole life-cycle of a stream-dwelling trout species. It is structured in nine processes: one related to the reach and cells (update of environmental and habitat conditions), five concerning trout (habitat selection, feeding and growth, survival, reproduction, and ageing) and three performed by redds (development, survival, and hatching of eggs and genetic transmission of traits to new trout).

The reach and cells update their state variables every time step over the whole simulation; trout perform each process every time step of the simulation, but for reproduction, which only occurs during the spawning season (every time step), and angling and hooking mortality, which is restricted to the angling season (every time step); trout age every time step but change their age-class once a year (the Julian day they were born); redd’s development and survival processes occur on a time-step basis since redd creation until all eggs have hatched; transmission of heritable traits occurs just when the egg hatches and the new trout is created.

*Schedule*: The simulation starts at an initial date set by the user through the input parameter *initial-date*. Environmental and habitat updates are scheduled first because subsequent trout and redd actions depend on the time step’s environmental and habitat conditions. Trout actions occur before redd’s because one trout action (reproduction) can cause redd mortality via superimposition. Reproduction is the first trout action because spawning can be assumed the primary activity of a fish on the day it spawns. Spawning also affects habitat selection because 1) spawners move to the spawning habitat when a redd is created and fertilized, and 2) spawners incur on weight, and thus body condition, loss after spawning, which affects their choice of habitat. Habitat selection is the second trout action each time step because it is the way that trout adapt to the new habitat conditions; habitat selection strongly affects both growth and survival. Feeding and growth precedes survival because changes in a trout’s length or condition factor affect its probability of survival. Survival has its own sub-schedule because the order in which survival probabilities for the different mortality sources are evaluated strongly affects the number of trout killed by each mortality source. Widespread, less random mortality sources are scheduled first: 1) high temperature, 2) high water velocity, 3) stranding, 4) poor condition, 5) predation by terrestrial animals, 6) predation by piscivorous fish, and 7) angling and hooking. The user has the possibility of choosing which mortality sources can kill trout during the simulation and which ones are not taken into account. Redd actions occur after cell and most trout actions because redds do not affect either habitat or fish, with the exception of creating new trout, which do not execute therefore their first actions until the day after their emergence. Redd survival is the first redd action to be executed. It includes five separate egg mortality sources that follow their own sub-schedule, from least to most random: 1) low temperature, 2) high temperature, 3) scouring, 4) dewatering, 5) superimposition. Trout emergence and genetic transmission of heritable traits is the last redd action. Since survival is scheduled before emergence, trout within redds are subject to redd mortality on the day they emerge (but not to trout mortality). Trout ageing is the last agent’s executed action each time step so that both pre-existent and new created trout can increase their age. Finally, observer actions (plotting graphs and writing output files) take place at the end of the time step. All actions occur in the same predetermined order:

1. Reach updates environmental and biological conditions. Cells update depth and velocity as a function of flow, and drift/search food production rate.

2. Trout reproduce:

2.1. Trout become spawners.

2.2. Trout spawn and create redds.

3. Trout select habitat.

4. Trout feed and grow: update length, weight and body condition factor.

5. Trout survive or die.

6. Redds’ eggs survive or die.

7. Redds’ eggs develop.

8. Redds’ eggs hatch, new trout are created and heritable traits are transmitted.

9. Trout age.

10. Observer plots model graphical outputs and write model output files.

## 1.4 Design concepts

Learning and Collectives concepts do not apply to this IBM.

*Basic principles*: The model was designed with an eco-genetic structure to analyze both ecological and genetic effects on population dynamics and life-history evolution on contemporary timescales. Accounting for inheritance of quantitative genetic traits allows the study of the eco-evolutionary responses of populations to changing environmental conditions, extreme climate events and strong anthropogenic selection pressures. InSTREAM-Gen is therefore a spatial dynamics model, which integrates the demographic, genetic and spatial dimensions of individual variability through its underlying spatially explicit bioenergetics model and its quantitative genetic model of inheritance of genetic traits.

InSTREAM-Gen is underpinned by "State and prediction-based theory", a new approach that combines existing trade-off methods with routine updating: individuals make a prediction of the future growth and risk conditions over an entire time horizon under different alternative behaviours, but each time they update their decision by considering how their internal state and external conditions have changed, so that they can select the alternative optimizing a fitness measure (see review by Railsback and Harvey 2013).

In inSTREAM-Gen, population abundance and structure can be influenced by density-dependent or density-independent processes. Direct density dependence is only represented through the aquatic predation mortality function, which partly depends on density of piscivorous fish in the reach. Indirect density-dependent mortality occurs during the spawning season, since increasing number of spawners increases the probability of redds dying by superimposition. Density-independent mortality factors include terrestrial predation, flow and temperature extreme events and recreational fishing.

*Emergence*: Dynamics of population demographics (abundance, biomass, production, age- and size-structure) and genetics (evolutionary changes in life-history traits such as size-at-emergence, size maturity threshold, age-at-first-reproduction, time of spawning and emergence) emerge from the growth, survival, and reproduction of individuals, individual-level processes which are driven by complex interactions between individuals and their spatio-temporally heterogeneous habitat. Likewise, other population-level responses, like density-dependent mortality and growth, and habitat selection patterns, are emergent properties of the modelled systems.

*Adaptation*: Habitat selection (i.e., the decision of which cell to occupy each time step) is the primary adaptive trait of trout, strongly driving trout growth and survival. Other adaptive trait is the selection of the feeding strategy (drift-feeding vs. search-feeding) a fish uses each time step, since it directly affects growth and, indirectly, survival. Trout are able to adapt some of their reproductive behaviors to environmental conditions and their own state: The decision by female spawners of when and where to spawn affects offspring production as well as recruitment survival and growth; selection of male spawners by female spawners is based on the male’s body condition factor and size, and offspring’s genotypic body size and size maturity threshold are inherited from their parents.

*Objectives*: Habitat selection is modelled as a fitness-seeking process, by which trout select the cell that maximizes “Expected Reproductive Maturity”, a fitness measure developed by Railsback et al. (1999) that represents the expected probability of surviving and reaching reproductive size over a future time horizon.

*Prediction*: Trout are able to predict the probability of both surviving starvation and other mortality sources (except fishing mortality), and approaching maturity size over a future time horizon defined by the user.

*Sensing*: Trout sense water temperature, which influences growth and survival, and consequently, habitat selection. Redds sense water temperature too, affecting survival, development and the timing of hatching. Trout perceive the cell’s habitat conditions, both hydraulic conditions and structural features (cover and substrate). This is a main driver of habitat selection. In the case of redds, they also sense their hydraulic environment, which determines the probability of survival of eggs. Trout are aware of all mortality sources in the model and are able to estimate the risk posed by each of them (but for fishing mortality, whose risk is not sensed).

*Interaction*: Competition for food and feeding habitat (velocity shelters) are modelled explicitly, at the cell scale, according to a size-based dominance hierarchy. Each habitat cell contains a limited daily food supply and a fixed area of velocity shelter, so that the food consumed and the sheltered area once used by larger trout are not available for smaller fish. Sexual selection is simulated by indirect interactions of males through their relative weight and condition factor.

*Stochasticity*: InSTREAM-Gen is not a highly stochastic model. The most important process represented as stochastic is trout and redd mortality. While mortality is modelled by calculating the daily probability of each individual agent’s survival through deterministic logistic functions, whether the agent actually lives or dies is a stochastic event. Stochasticity is also used in the reproduction process for setting the timing of redd creation, and for the selection of the number and identity of males fertilizing the eggs of a redd, as well as of the identity of the male spawner transmitting its genetic inheritance to each egg. The genotypic and phenotypic values of heritable traits of new created trout are drawn from empirical probability distributions. Likewise, position, sex, age (in days), body size, as well as the genotypic value and its phenotypic expression of heritable life-history traits of trout at initialization are stochastic (drawn from probabilistic functions).

*Observation*: The model produces both graphical displays and output files.

The model provides a graphical display of habitat cells and the location of fish and redds as the model executes. In addition, the model provides several graphical displays of model outputs: population structure updated on a tick basis; fish numbers and biomass, dead fish numbers broken out by mortality source, and total number of eggs in the reach, all updated on a tick basis; yearly demographic outputs (written on the Julian date set by *OutputDate* parameter) including fish numbers and biomass, dead fish numbers broken out by mortality source, number of breeders, and number of initial eggs and fry hatched; yearly life-history outputs including minimum, mean and maximum values of length-at-age, length and age at spawning broken out by sex, spawning date, emergence date, and age at death; yearly genetic outputs including minimum, mean and maximum values of genotypic length at emergence, neutral trait and length maturity threshold, the latter broken out by sex.

The following demographic and genetic outputs can be recorded at the population level to follow the changes through time of the population ecogenetic structure: 1) Summary population statistics (*LiveFishOutput* file): These statistics include abundance, abundance of mature fish, total fish biomass, and mean and variability (standard deviation) of fish length, weight, and phenotypic values of length maturity threshold, length at emergence and neutral trait, broken out by age-class; 2) Summary breeder population statistics (*BreedersPopOutput* file): These statistics include abundance, and mean, minimum value and variability (standard deviation) of age and fish length at spawning, all broken out by sex. It includes also both phenotypic and genotypic values of length at emergence, neutral trait and length maturity threshold, the latter broken out by sex. Finally, the output file records the mean, minimum value and standard deviation of spawning date and date of emergence of the offspring; 3) Fish mortality (*DeadFishOutput* file): It records the number of fish that have died of each mortality source during a time step, broken out by age class; 4) Redd status and mortality (*ReddOutput* file): It reports when a redd was created, how many viable eggs were created, and when the redd was removed from the model because all its eggs had died or emerged, together with the number of eggs died from each redd mortality source and the number of emerged new trout.

The model also allows the possibility of recording life-history features of breeders at the individual level (*BreedersIndOutput* file): It records the trout and fertilized redd’s IDs, the sex, age-class, age, length and weight at spawning, as well as both the phenotypic and genotypic values of length maturity threshold, length at emergence and neutral trait. Habitat use and availability can be recorded through the *HabSelecOutput* file: It reports, for every cell, its area, depth, velocity, fraction with velocity shelters, fraction with cover from predation, average distance to hiding cover, and food availability (drift and search food production rates, as well as the number of trout in the cell broken out by age-class. The output file also provides the flow, temperature and total trout abundance in the reach.

Both demographic fish output files (*LiveFishOutput* and *DeadFishOutput*) can be either written on a yearly or tick basis (set through the *AnnualFishOutput?* global parameter). When written on a tick basis, the parameter *fileOutputFreq* sets the frequency. The *OutputDate* parameter defines the Julian date when the yearly population outputs are written, but for the *ReddOutput* file, which is updated every time a redd is dead or emptied.

## 1.5 Initialization

At initializing a model run, the user must specify the initial date of simulation and the duration of a time step. State of reach’s environmental variables, as well as cells’ hydraulic and habitat variables are input data. Trout population numbers, age-structure and length-distribution are input data. Population-level distributions of heritable traits are also input data.

Each individual’s state variable (sex, age, length, and genotypic and phenotypic values of heritable traits) is initialized by drawing from probability distributions describing their variability. Length and genotypic values of heritable traits are truncated at 4 standard deviations from the center of the probability distributions. Length of 0+ trout cannot be lower than a minimum user-defined value *fishMinNewLength*. Trout weight is calculated as a function of length:

*fishWeight* = *fishWeightParamA* × (*fishLength*)^fishWeightParamB^ (1)

and condition factor is subsequently calculated as a function of body length and weight. The condition factor variable used in the model (*fishCondition*) can be considered the fraction of “healthy” weight a fish is, given its length (approach adopted from Van Winkle et al. 1996). The value of *fishCondition* is 1.0 when a fish has a “healthy” weight for its length, according to the length-weight relationship. Trout maximum sustainable swimming speed is a function of the fish’s length and water temperature. It is modelled as a two-term function, where the first term represents how it varies linearly with fish length, while the second modifies maximum swimming speed with a non-linear function of temperature:

(2)

*fishMaxSwimSpeed* [cm s^-1^] = [*fishMaxSwimParamA* × *fishLength* + *fishMaxSwimParamB*]

× [*fishMaxSwimParamC* × (*temp*)^2^ + *fishMaxSwimParamD* × *temp* + *fishMaxSwimParamE*)]

Status is set to “alive”. Maturity status is set to either “mature” or “non-mature” depending on whether trout’s initial length is over or under the phenotypic value of the length maturity threshold (*fishSpawnMinLength*). The *spawnedThisSeason?* variable is set to “NO”.

Each trout’s location is assigned stochastically while avoiding extremely risky habitat. The model limits the random distribution of trout to cells where the trout are not immediately at high risk of mortality due to high velocity or stranding. Therefore, each trout is located in a random wetted cell (*cellDepth* > 0) with a ratio of cell velocity to the trout’s maximum swimming speed (*cellVelocity* / *fishMaxSwimSpeed*) lower than the parameter *mortFishVelocityV9*, the value at which the probability of surviving high velocity mortality equals 0.9 (see 1.7 Submodels Section 5).

## 1.6 Input data

Times series of three reach environmental variables (temperature, flow and Julian date) are input data. Temporal series of cells’ hydraulics (water depth and velocity) are input data too. Fixed physical habitat features of cells (spatial location, and fraction of the cell’s area having velocity shelters, elements providing cover from predators and gravels) are specified by means of input files.

## 1.7 Submodels

Since the demographic structure of inSTREAM-Gen is a replicate of inSTREAM IBM, the formulation of all its submodels follows the approaches and equations originally developed by and described in Railsback et al. (2009), unless it is otherwise explicitly stated.

*1.7.1. Environmental and habitat conditions update*:

1.7.1.1. Reach updates temperature and flow from input time series. Day length is calculated and updated:

$dayLength=24-2\left[ \left( \frac{12}{\pi} \right)\mathrm{arcos}\left\{ \tan\left( \frac{\pi\times siteLatitude}{180} \right)\tan\delta\right\} \right]$ (3)

where $\delta=\left[ \left( \frac{23.45}{180} \right)\pi cos\left\{ \left( \frac{2\pi}{365} \right)(173-julianDate) \right\} \right]$ (4)

1.7.1.2. Cells update hydraulics (depth and velocity) from input time series. *wettedArea* and *cellDistanceToHide* are calculated accordingly. *wettedArea* is simply the sum of the area of all cells with *cellDepth* > 0. *cellDistanceToHide* represents the average distance a fish located in the cell would need to move to find hiding cover. While in inSTREAM, *cellDistanceToHide* is fixed along time, in inSTREAM-Gen it dynamically changes its value every time step. Importantly, cover is only available if the covered cell is not dry; in a cell with cover, it is the average distance within the area with cover (0 meters) plus the average distance within the area without cover (represented as a circumference); in a cell without cover, it is the sum of the distance to the closest covered cell plus the average distance within the area without cover of the closest covered cell.

1.7.1.3. Production rates of both drift and search (stationary) food in the cell are updated. Importantly, the trout feeding submodel uses hourly food production and consumption rates because the number of feeding hours per day varies.

Drift food production rate is calculated from hydraulic data, being modelled as the rate at which prey items flow into the cell from upstream, plus the rate at which consumed prey are regenerated within the cell:

*driftHourlyCellTotal* [g h^-1^] = 3600 [s h^-1^] × *cellDepth* [cm] × *cellVelocity* [cm s^-1^] (5)

× *cellArea* [cm^2^] × *habDriftConc* [g cm^-3^] / *habDriftRegenDist* [cm]

where *habDriftConc* and *habDriftRegenDist* are reach parameters representing the drift food density in the reach and the drift regeneration distance, respectively.

The rate at which search food is produced in a cell is simply the cell area multiplied by a reach parameter defining the search food density rate:

*searchHourlyCellTotal* [g h^-1^] = *habSearchProd* [g cm^-2^ h^-1^] × *cellArea* [cm^2^] (6)

1.7.1.4. The density of piscivorous fish (*PiscivFishDens*) is calculated as the number of trout with a fish length greater than the reach parameter *fishPiscivoryLength* divided by the reach’s wetted area (*wettedArea*). The value of the temperature function of the trout’s physiological maximum daily food consumption (*cmaxTempFunction*) is updated as a function of updated water temperature.

*1.7.2. Trout Reproduction and Redd creation*:

It is scheduled in two main actions:

1.7.2.1. Trout become spawners:

Every day, each female trout determines whether to spawn based on whether it meets all of the following fish- and habitat-based spawning criteria:

- Trout only spawn within a spawning date window (spawning season) defined by the global parameters *fishSpawnStartDate* and *fishSpawnEndDate*.

- Trout have to be sexually mature (maturity is attained when the trout reaches an age and length equal to *fishSpawnMinAge* and *fishSpawnMinLength*, respectively) and have enough energy reserves to spawn (its condition factor must exceed the minimum condition factor parameter *fishSpawnMinCond*).

- Female trout are assumed not to spawn more than once per annual spawning season. At the start of the first day of the spawning season the Boolean variable *spawnedThisSeason?* is set to NO for all trout. Once a female trout spawns, the variable is set to YES so that the trout is not allowed to spawn again during the rest of the spawning season.

- Trout only spawn within a temperature range defined by parameters for maximum and minimum temperatures for spawning (*fishSpawnMaxTemp* and *fishSpawnMinTemp*, respectively).

- Trout cannot spawn if the flow in the reach is higher than a maximum threshold defined by the reach habitat parameter *habMaxSpawnFlow*.

- Trout are assumed not to spawn when flows are unsteady. Therefore, if the fractional change in flow from the previous day is greater than the value of the parameter *fishSpawnMaxFlowChange* then spawning is not allowed. This fractional change in flow is evaluated as:

*fracFlowChange* = abs(*todaysFlow* - *yesterdaysFlow*)/*todaysFlow*  (7)

Finally, on the time-steps when all the spawning criteria are met for a female, then whether it actually spawns is determined stochastically. The probability of spawning on any such day is the parameter *fishSpawnProb* (unitless).

A male trout becomes spawner, only within the spawning season, when it is sexually mature (its age and length are equal or greater than *fishSpawnMinAge* and *fishSpawnMinLength*, respectively) and has a body condition over *fishSpawnMinCond*. Males are able to spawn multiple times over the spawning season, as it is typically described in the literature (Jonsson and Jonsson 2011).

1.7.2.2. Redd creation and fertilization:

This action assumes a size-based dominance hierarchy for spawning, so that the following steps are carried out in descending order of fish length.

1.7.2.2.1. Selection of the spawning cell.

Female spawners select the cell in which they then build a redd. The first step in identifying the location for a new redd is identifying all the cells that are potential spawning sites. It follows the same method used by trout to identify potential destinations during habitat selection (1.7 Submodels Section 3). Afterwards, potential spawning cells are rated by the spawner to identify the cell with the highest value of variable *spawnQuality*:

*spawnQuality* = *spawnDepthSuit* × *spawnVelocitySuit* × *spawnGravelArea*  (8)

where variables *spawnDepthSuit* and *spawnVelocitySuit* are unitless habitat suitability factors, whose values are interpolated linearly from suitability functions provided as parameters. The value of *spawnGravelArea* is the cell area times its fraction with spawning gravel (*cellArea* × *cellFracGravel*). If *spawnGravelArea* is 0 then the female trout moves to the cell that maximizes (*spawnDepthSuit* × *spawnVelocitySuit*).

The female trout moves then to the selected spawning cell to create a redd.

1.7.2.2.2. Selection of male spawners.

InSTREAM-Gen allows for both monogamy (each cross involving two parents) and polygamy (each cross involving one female and several satellite males) mating strategies. Both monogamous and polygamous matings have been commonly observed in trout breeding systems (García-Vázquez et al. 2001, Serbezov et al. 2010a). The number of males per female (*number-males*) is randomly drawn from a uniform distribution from 1 to *max-n-males-per-female* (a global parameter). Following Piou and Prévost (2012), the probability of a male spawner *j* of being selected to fertilize a redd depends on its weight:

P (selected│ *fishWeight*_j_) = (*fishWeight*_j_ / ∑ *fishWeight*_l_) (9)

where *l* is the number of available male spawners. The largest male is always selected. The rest of *number-males* males are then randomly selected among a list containing the *n* male spawners having the highest probability (*candidate-spawners*). This number *n* is stochastically chosen. If the number of selected spawners is lower than *number-males*, then additional males are randomly selected (if possible) among the remaining male spawners until *number-males* is reached. If no male meets the criteria as a spawner, or there are no more male spawners available, there is no effect on the female or redd and the female still produces a fertile redd, so that transmission of heritable traits depends only on the mother's genotypic values.

Contrarily to inSTREAM, male spawners move to the spawning cell selected by the female.

1.7.2.2.3. Redd creation and fertilization.

- When a female spawner has selected a spawning cell, it creates a redd in the cell. The number of eggs in the redd depends on the spawner’s fecundity (a function of length) and losses during spawning:

*numberOfEggs* = (*eggsize-fecund-tradeoff* × *fishFecundParamA* × *fishLength^fishFecundParamB^)*

× *fishSpawnEggViability* (10)

Since trout length at emergence is a heritable trait in the model and it is typically correlated to egg size (see references in reviews by Klemetsen et al. 2003, and Jonsson and Jonsson 2011), we introduced the term *eggsize-fecund-tradeoff* to deal with the fact that in salmonids the number of eggs in a redd is traded-off with egg size (see again Klemetsen et al. 2003, Jonsson and Jonsson 2011). It was modelled as the relationship between the number of eggs that would be created by the trout if the offspring had the population mean length at emergence and such number if the offspring had the female spawner's genetic length at emergence, which is mathematically expressed as:

*eggsize-fecund-tradeoff* = (*fishNewLengthMean* / *genNewLength*)*^fishWeightParamB^* (11)

The parameter *fishSpawnEggViability* is the fraction of eggs that are successfully fertilized and placed in the redd.

- After the redd is created by the female trout, it is fertilized by the selected male spawners. That means that the genotypic value of heritable traits of both the mother and all fathers are stored in the genetic trait map of the redd (*reddMothergenSpawnMinLength*, *reddMotherNewLength*, *reddMotherNeutralTrait; reddFathersgenSpawnMinLength*, *reddFathersNewLength*, *reddFathersNeutralTrait*).

1.7.2.2.4. Incur weight loss.

Both female spawner and all males contributing to the redd incur on weight loss. Their weight is reduced according to the parameter *fishSpawnWtLossFraction*, so that *fishWeight* is multiplied by (1- *fishSpawnWtLossFraction*). In consequence, the body condition factor is accordingly reduced, which can significantly affect subsequent habitat selection and survival.

*1.7.3. Trout Habitat selection*:

The habitat selection trait is modelled as follows: every time step, each trout moves to the habitat cell that (1) is close enough that the fish can be assumed to be aware of conditions in it, and (2) offers the highest “expected fitness”, where expected fitness is approximated as the expected probability of surviving and reaching reproductive size over a future time horizon.

The habitat selection trait assumes a size-based dominance hierarchy: trout can only use resources (food and velocity shelters) that have not been consumed by larger trout. The number of trout feeding in a cell is limited by its daily food production. Each trout using a drift-feeding strategy can use a maximum velocity shelter area (cm^2^) equal to (*fishLength*)^2^.

1.7.3.1. Identify potential destination cells:

When each individual trout begins its habitat selection procedure, its first action is to identify the cells that are potential movement destinations. Distance and depth can limit potential destination cells, but the number of fish already in a cell does not limit its availability as a destination.

Only wetted (*cellDepth* > 0) habitat cells within a certain distance are included as potential destinations. This maximum movement distance should be considered the distance over which a fish is likely to know its habitat well enough to be aware when desirable destinations are available, over the time step. It is an exponential function of fish length (Diana et al. 2004):

*maxMoveDistance* (cm) = *fishMoveDistParamA* × (*fishLength*) *^fishMoveDistParamB^* (12)

However, as discussed by Railsback et al. (2009), for small fish, it is possible that no cells other than the current one are within this *maxMoveDistance*, which poses an artificial barrier to movement, an artifact of the model’s spatial resolution. Consequently, a fish’s potential destinations always include the cells adjacent to the fish’s current cell.

1.7.3.2. Evaluate potential destination cells:

A trout evaluates each potential destination cell to determine the fitness it would provide, using the “Expected Reproductive Maturity” fitness measure of Railsback et al. (1999), where:

*expectedMaturity* = *nonstarvSurvival* × *starvSurvival* × *fracMature* (13)

*nonstarvSurvival* is the probability of survival for all mortality sources except starvation and angling and hooking over the fitness horizon (see Submodels Section 5); its formulation implicitly assumes that trout consider all mortality sources in their habitat selection decision. This means that the trout are assumed to be aware of all the kinds of mortality in the model and are able to estimate the risk posed by each, except for fishing mortality:

*nonstarvSurvival* = (*S_hightemp_* × *S_highvel_* × *S_strand_* × *S_terrpred_* × *S_aqpred_*)*^fishFitnessHorizon^* (14)

*starvSurvival* represents the probability of surviving starvation over the fitness horizon; the method assumes that trout evaluate expected maturity using the simple prediction that the current time-step’s growth rate would persist over the time horizon. It is implemented following the next steps:

First, determine the foraging strategy, food intake, and growth for the trout and habitat cell in question, for the current time step, using the methods described in Section 1.7.4.

Second, project the fish’s weight, length, and condition factor that would result if the current day’s growth persisted over the fitness time horizon specified by *fishFitnessHorizon*.

Third, approximate the probability of surviving starvation over the fitness horizon, estimated as the first moment of the logistic function of poor condition survival vs. condition factor:

$starvSurvival = \left[ \frac{\left( \frac{1}{b} \right)\ln\left( \frac{1+e^{(a + b \times K_{t+T})}}{1+e^{(a + b \times K_{t})}} \right)}{\left( K_{t+T}-K_{t} \right)} \right]^{T}$ (15)

where *K*_t_ is the fish’s value of *fishCondition* at the current time-step and *K*_t+T_ is the projected condition factor at the end of the fitness horizon, *T* is equal to *fishFitnessHorizon*, and *a* and *b* are the *logistA* and *logistB* variables of the logistic function of poor condition survival [described in Section 1.7.5, equation (42)].

This equation can cause significant computational errors when *K_t_*_+_*_T_* is extremely close to *K_t_* (and a divide-by-zero error when they have the same value). To avoid it, *starvSurvival* is set equal to the daily survival probability for *Kt*, raised to the power *fishFitnessHorizon*, whenever (*K_t_*_+_*_T_* - *K_t_*) is less than 0.001.

*fracMature* represents how close to the size of sexual maturity a fish would be at the end of the fitness time horizon. It is simply the ratio between (a) the length the fish is projected to be at the end of the time horizon, and (b) the parameter *fishSpawnMinLength* (see Section 1.7.2), limited to a maximum of 1.0.

*1.7.4. Trout Feeding and Growth*:

In the model, trout can use either of two feeding strategies, drift or active search feeding. The feeding and growth methods calculate the potential food intake and metabolic costs a fish would experience in a cell, for both drift and search feeding. Standard bioenergetics approaches are used to calculate net energy intake (the difference between energy intake from food and metabolic energy cost) for each feeding strategy (following Hanson et al. 1997). The fish then selects the strategy that provides the highest net energy intake. Daily growth is proportional to net energy intake. A fish’s length and condition factor at the end of the time-step are updated from its daily growth. The following steps describe the process used by a trout to determine the feeding strategy it would use, and the resulting food intake and growth it would obtain, for a particular habitat cell.

1.7.4.1. Feeding:

1. Determine the **potential daily drift intake** that would be obtained in the absence of more dominant fish in the cell. This *dailyPotentialDriftFood* is determined from the hourly intake rates and hours spent feeding:

*dailyPotentialDriftFood* [g d^-1^] = *driftIntake* [g h^-1^] × *feedTime* [h d^-1^] (16)

Hours spent feeding is the day length plus one hour before sunrise and one after sunset:

*feedTime* = *dayLength* + 2

A fish’s intake rate is calculated as the mass of prey passing through the capture area times the capture success:

*driftIntake* [g h^-1^] = *habDriftConc* [g cm^-3^] × *cellVelocity* [cm s^-1^] × *captureArea* [cm^2^] (17)

× 3600 [s h^-1^] × *captureSuccess* [unitless]

The capture area models the area over which drift-feeding trout can detect prey and is depicted as a rectangular area perpendicular to the current, whose dimensions ultimately depend on fish size through the detection distance. Fish are assumed able to detect all drift that comes within the detection distance to their left and right, while the height of the capture area is the minimum of the reactive distance and the depth (which often is lower):

(18)

*captureArea* [cm^2^] = [2 × *detectDistance* [cm]] × [min (*detectDistance*, *cellDepth*) [cm]]

Detection distance is defined as the distance over which fish can see and attack - but not necessarily capture - prey. Detection distance is primarily a function of the size of the fish:

(19)

*detectDistance* (cm) = *fishDetectDistParamA* + *fishDetectDistParamB* × *fishLength* [cm]

Railsback et al. (2009) developed this model based on empirical data from the study of Schmidt and O’Brien (1982) for arctic grayling, whose results had been used successfully as the basis of the previous drift feeding models of Hughes (1992a) and Hughes et al. (2003). The linear model is not, however, a regression fit to those data, but rather it was derived from pre-calibration of the growth model. In fact, an exponential model provided a better fit to the data, but the linear model, nevertheless, was able to capture a series of qualitative patterns the exponential formulation was not (see Railsback et al. 2009 or the “Data evaluation” element of the present TRACE document for further details).

Capture success represents what fraction of detected prey is actually caught. Capture success is largely a function of water velocity but also of the fish’s maximum sustainable swimming speed:

*captureSuccess* ~ logistic (*cellVelocity* / *fishMaxSwimSpeed*) (20)

Maximum sustainable swimming speed is a component of not only the drift feeding trait but also of high velocity mortality (Submodels Section 5.2), and strongly affects the relationship between a cell’s velocity and habitat quality for various size trout. The maximum swim speed used for both drift-feeding and high velocity mortality must be a speed that fish can swim for hours, not a burst or short-term maximum speed. It is a function of a fish’s length and water temperature, as described in equation (2).

2. Determine the **daily drift intake available** after more dominant fish in the cell have consumed their intake:

*dailyAvailableDriftFood* [g d^-1^] = *driftHourlyCellAvail* [g h^-1^] × *feedTime* [h d^-1^] (21)

The drift food production rate in a cell *driftHourlyCellTotal* is updated every time a trout moves to that cell, so that the drift food available in a cell for a trout is the drift food production rate in the cell at the beginning of the time-step minus the *driftIntake* of all larger trout using a drift-feeding strategy that already occupy the cell. Therefore, hierarchical competition for food is implemented via the food availability rates.

*driftHourlyCellAvail* [g h^-1^] = *driftHourlyCellTotal* – ∑ *driftIntake* (22)

3. Determine the **physiological maximum daily consumption (*cMax*)**:

Maximum daily consumption (*cMax*) represents the maximum rate of food consumption if a fish is limited only by its physiology. The equation for *cMax* includes (a) an allometric function, relating *cMax* to fish size; and (b) a temperature function (Hanson et al. 1997), which is represented as a set of seven points used to interpolate a value of *cmaxTempFunction* from the reach’s temperature:

*cMax* (g d^-1^) = *fishCmaxParamA* × (*fishWeight*)^(1 + fishCmaxParamB)^ × *cmaxTempFunction* (23)

4. Calculate the actual **daily drift food intake**, considering whether it is limited by actual food availability or the physiological maximum intake:

(24)

*dailyDriftFoodIntake* [g d^-1^] = min(*dailyPotentialDriftFood*, *dailyAvailableDriftFood*, *cMax*)

5. Convert daily drift intake in grams of food to joules of **energy** by means of the *Prey energy density* reach parameter:

(25)

*dailyDriftEnergyIntake* [j d^-1^] = *dailyDriftFoodIntake* [g d^-1^] × *habPreyEnergyDensity* [j g^-1^]

6. Conduct the bioenergetics energy balance to get **net energy intake for drift feeding**:

*dailyDriftNetEnergy* [j d^-1^] = *dailyDriftEnergyIntake* [j d^-1^] - *respTotal* [j d^-1^] (26)

The model uses the Wisconsin Model equation 1 for respiration (Hanson et al. 1997), as modified by Van Winkle et al. (1996) to apply the activity respiration rate only during active feeding hours. Respiration is therefore modelled as the energetic cost of metabolism and swimming, including then (a) standard respiration that is independent of the fish’s activity, and (b) an additional activity respiration that increases with the daily swimming speed.

Drift-feeding fish are assumed to swim at a speed (*swimSpeed*, cm s^-1^) equal to their habitat cell’s water velocity unless they have access to velocity shelter. If a drift-feeding fish has access to velocity shelter, then its *swimSpeed* is assumed equal to a constant fraction of its habitat cell’s mean water velocity, defined by the reach parameter *habShelterSpeedFrac*. A fish has access to velocity shelter in a cell only if the sum of shelter areas occupied by larger drift-feeding fish in the cell (each drift-feeding fish is assumed to use up an area of velocity shelter equal to the square of its length) is less than the cell’s total shelter area.

*respTotal* [j d^-1^] =*respStandard* [j d^-1^] + *respActivity* [j d^-1^] (27)

*respStandard* = (*fishRespParamA* × (*fishWeight*)^fishRespParamB^) × e^(^*^fishRespParamC^* ^× temp)^ (28)

*respActivity* = (*feedTime* / 24) × (e^(fishRespParamD × swimmSpeed)^ - 1) × *respStandard* (29)

7. Determine the **potential daily search feeding intake** that would be obtained in the absence of more dominant fish in the cell:

*dailyPotentialSearchFood* [g d^-1^] = *searchIntake* [g h^-1^] × time feeding [h d^-1^] (30)

The model assumes that the rate of search food intake is proportional to the rate at which search food becomes available: every fish searches for food at about the same rate, so intake increases linearly with food production. Search feeding intake is also assumed to decrease linearly to zero as water velocity increases to the fish’s maximum sustainable swim speed. This velocity function represents how the ability of a fish to see and search for food decreases with velocity. The rate of search food intake is formulated as follows:

*searchIntake* [g h^-1^] = *habSearchProd* [g cm^-2^ h^-1^] × *fishSearchArea* [cm^2^] (31)

× max([(*fishMaxSwimSpeed* – *cellVelocity*) / *fishMaxSwimSpeed*], 0)

where *habSearchProd* is the rate at which search food is produced, *fishMaxSwimSpeed* is the fish’s maximum sustainable swimming speed, and *cellVelocity* is the velocity of the fish’s cell. The proportionality constant *fishSearchArea* can be loosely interpreted as the area over which the production of stationary (non-drifting) food is consumed by one fish.

8. Determine the **daily search intake available** after more dominant fish have consumed their intake:

*dailyAvailableSearchFood* [g d^-1^] = *searchHourlyCellAvail* [g h^-1^] × *feedTime* [h d^-1^] (32)

In the same way that drift feeding is modelled, search food available in a cell for a trout is calculated as the search food production rate in the cell at the beginning of the time-step minus the *searchIntake* of all larger trout using a search-feeding strategy that already occupy the cell. Again, hierarchical competition for food is implemented via the food availability rates.

*searchHourlyCellAvail* [g h^-1^] = *searchHourlyCellTotal* – ∑ *searchIntake* (33)

9. Calculate the actual **daily search intake** considering whether it is limited by food availability or maximum daily intake:

(34)

*dailySearchFoodIntake* [g h^-1^] = min(*dailyPotentialSearchFood*, *dailyAvailableSearchFood*, *cMax*)

10. Convert daily search intake in grams of food to joules of **energy** by means of the *Prey energy density* reach parameter:

(35)

*dailySearchEnergyIntake* [j d^-1^] = *dailySearchFoodIntake* [g d^-1^] × *habPreyEnergyDensity* [j g^-1^]

11. Conduct the bioenergetics energy balance to get net energy intake for search feeding:

*dailySearchNetEnergy* [j d^-1^] = *dailySearchEnergyIntake* [j d^-1^] – *respTotal* [j d^-1^] (36)

Respiration costs for fish using a search feeding strategy are calculated in the same way than for drift-feeding fish. However, fish using the search feeding strategy are assumed to swim at a speed equal to their cell’s mean water velocity. There is no reduction in *swimSpeed* due to velocity shelters.

12. Select the most profitable feeding strategy by comparing *dailyDriftNetEnergy* to *dailySearchNetEnergy*; and determine the **energy intake for the best strategy**:

*bestNetEnergy* [j d^-1^] = max(*dailyDriftNetEnergy*, *dailySearchNetEnergy*) (37)

2.7.4.2. Growth:

13. Convert net energy intake to **daily growth** by means of the *Fish energy density* reach parameter:

*dailyGrowth* [g d^-1^] = *bestNetEnergy* [j d^-1^] / f*ishEnergyDensity* [g j^-1^] (38)

14. Update the fish’s **weight** at the end of the time-step:

*FishWeight* [g] = *fishWeight* [g] + *dailyGrowth* [g d^-1^] × *timestep-scale* [d] (39)

15. Update the fish’s **length** at the end of the time-step:

Fish length is then the maximum length between current length and potential length (*fishWannabeLength*; the length the fish would be if its condition factor were 1.0). This potential length is calculated with the fish’s new weight and the inverted length-weight relation for healthy fish:

*fishWannabeLength* [cm] = (*fishWeight* / *fishWeightParamA*)^(1 /^ *^fishWeightParamB)^* (40)

If the fish’s current length is less than *fishWannabeLength* (indicating that the fish is not underweight), then its new length is set to *fishWannabeLength*. Otherwise, its length is not changed.

16. Update the fish’s **condition factor** at the end of the time-step:

The new value of *fishCondition* is equal to the fish’s new weight divided by the “healthy” weight for a fish given its length:

*fishCondition* = *fishWeight* / (*fishWeightParamA* × (*fishLength*)*^fishWeightParamB^*) (41)

*1.7.5. Trout Survival*:

Survival simulations determine, each day, which fish die from what causes. Mortality sources are represented separately because the probability of surviving each varies differently with fish state and habitat conditions. Mortality sources are represented as survival probabilities: the daily probability of not being killed by one specific mortality source. Survival probabilities are used (1) during habitat selection (Section 1.7.3) as a major input trout use in deciding which habitat cell to occupy, and (2) to model mortality: when and why each fish actually dies. The same methods are therefore used to determine survival probabilities in modelling both habitat selection and mortality.

On every simulated time step, each fish determines whether it dies of each mortality source following a two-step process: first is calculating the daily survival probability from the current state of the fish and its cell and project it over the time extent defined by *timestep-scale*; second is determining, stochastically, whether the fish actually dies by comparing a random number drawn from a uniform distribution between zero and one to the projected survival probability. If the random number is greater than the survival probability, then the fish dies as a result of the mortality source and no further mortality sources are evaluated for the fish. If the fish does not die, then the next mortality source is evaluated. The user has the option to select the mortality sources that can actually kill the trout.

The survival probabilities are modelled through logistic functions, so that their values increase from zero to one, or decrease from one to zero, along the range of the predictor used as a proxy for the evaluated mortality source. In the model, logistic functions are defined via parameters that specify the predictor values at which the survival probability value equals 0.1 and 0.9. The logistic functions are defined as:

*S* = e*^Z^* / (1 + e*^Z^*) (42)

where

*Z* = *LogistA* + (*LogistB* × *habitatVariable*),

*LogistA* = *LogistC* – (*LogistB* × *habVarAtS01*),

*LogistB* = (*LogistC* – *LogistD*) / (*habVarAtS01* – *habVarAtS09*),

*LogistC* = ln(0.1/0.9), and

*LogistD* = ln(0.9/0.1).

While death due to each mortality source is treated independently, the order in which mortality sources are evaluated can have a (usually very small) effect on how many fish die of each kind of mortality. They are scheduled in the following order:

1.7.5.1. High temperature:

This mortality source represents the breakdown of physiological processes at high temperatures. It does not represent the effect of high temperatures on bioenergetics (reduced growth at high temperature). The survival probability is based on the daily mean water temperature.

1.7.5.2. High velocity:

The high velocity survival function represents the potential for trout to suffer fatigue or lose their ability to hold position in a cell with high velocity. This function is included not because trout often die due to high velocity, but because it strongly affects habitat selection: mortality due to high velocities is not observed in nature because fish avoid it by moving. The survival probability is based on the ratio of the swimming speed a fish uses in a cell to the fish’s maximum sustainable swim speed (described in Section 1.7.4.1).

1.7.5.3. Stranding:

Stranding mortality represents the death of fish that are unable to move out of cells that become extremely shallow or dry as flow decreases. Survival of stranding is modelled as an increasing logistic function of depth divided by fish length in order to scale how the risks of low depths vary with fish size.

1.7.5.4. Poor condition:

Fish in poor condition (low value of the condition factor, weight in relation to length) are at risk of starvation, disease, and excess vulnerability to predators. These risks are combined in the poor condition survival probability. Poor condition can have a strong effect on habitat selection as well as mortality. As commented, the survival probability is based on the fish’s condition factor.

1.7.5.5. Terrestrial predation:

The formulation of this mortality source assumes a minimum survival probability *mortFishTerrPredMin* that applies when fish are most vulnerable to terrestrial predation, and a number of “survival increase functions” that can increase the probability of survival above this minimum. Survival increase functions are described as logistic functions that have values between zero and one, with higher values for greater protection from predation. The survival increase functions are assumed to act independently. Therefore, the terrestrial predation survival probability is obtained by increasing the minimum survival (decreasing the difference between minimum survival and 1.0) by the maximum of the independent survival increase functions. This assumption is expressed mathematically as:

(43)

*terrPredSurv* = *mortFishTerrPredMin* + [(1 – *mortFishTerrPredMin*) × max(*terrPredDepthF*, *terrPredLengthF*, *terrPredFeedTimeF*, *terrPredVelF*, *terrPredCoverF*)]

where *terrPredDepthF* is the value of the survival increase function for **depth**. The depth survival increase function is an increasing logistic curve: survival increases as depth increases; *terrPredLengthF* is the value of the survival increase function for **fish length**. Survival of terrestrial predation is assumed to decrease with fish length; *terrPredFeedTimeF* is the value of the survival increase function for **feeding time**. The survival increase function is modelled as a decreasing function of *feedTime* (h), the hours spent feeding per day; *terrPredVelF* is the value of the survival increase function for **water velocity**. The survival increase function is an increasing logistic curve: survival increases with velocity; *terrPredCoverF* is the value of the survival increase function for **distance to hiding cover**. Hiding cover is represented with a survival increase function that increases as distance to hiding cover (*cellDistanceToHide*, cm) decreases.

1.7.5.6. Aquatic predation:

The aquatic predation formulation represents mortality due to predation by fish. By adjusting parameter values, the formulation can be made to apply both to sites where the modelled trout are the only piscivorous fish and sites where non-trout fish, not otherwise represented in the model, are a significant risk. The formulation can represent the effect of adult trout density on aquatic predation survival, making this survival probability the only component of the model with direct density dependence. It allows a type of feedback that is potentially important in regulating trout populations: when adult abundance is greatly reduced, juveniles can safely use a wider range of habitat and, hence, have greater growth and survival to adulthood.

As with terrestrial predation, the formulation uses a minimum survival probability *mortFishAqPredMin* that applies when fish are most vulnerable to aquatic predation, and a number of survival increase functions:

(44)

*aqPredSurv* = *mortFishAqPredMin* + [(1 – *mortFishAqPredMin*) × max(*aqPredDensF*, *aqPredDepthF*, *aqPredLengthF*, *aqPredFeedTimeF*, *aqPredTempF*)]

where *aqPredDensF* is the value of the survival increase function for **piscivorous trout density**. This function represents only the effect of trout included in the model and not of other piscivorous fish that may be present in the reach. Any trout with length greater than the parameter *fishPiscivoryLength* (cm) is assumed to be a piscivorous trout. Predation is represented at the reach spatial scale (as opposed to the cell scale) because large, piscivorous trout are likely to foray and attack fish in other cells. The predator density survival increase function causes the survival increase function to increase as the density of piscivorous trout decreases; *aqPredDepthF* is the value of the survival increase function for **depth**. The depth survival increase function is a decreasing logistic function; *aqPredLengthF* is the value of the survival increase function for **fish length**. Survival of aquatic predation is increases with fish length; *aqPredFeedTimeF* is the value of the survival increase function for **feeding time**. The survival increase function is modelled as a decreasing function of the hours spent feeding per day; *aqPredTempF* is the value of the survival increase function for **water temperature**. This survival increase function reflects how low temperatures reduce the metabolic demands and, therefore, feeding activity of piscivorous fish. The survival increase function is therefore a decreasing logistic curve.

*1.7.6. Redd Survival*:

Eggs incubating in a redd are subject to five mortality sources: low and high temperatures, scouring by high flows, dewatering, and superimposition (having another redd laid on top of an existing one). Redd survival is modeled using redd “survival functions”, which determine, for each redd on each day, the probability of each egg surviving one particular kind of mortality. Then, a random draw is made on a binomial distribution to determine how many eggs survive each redd mortality source. The binomial distribution returns a randomly drawn number of eggs that die each day, given the number of live eggs in the redd and the per-egg mortality probability (one minus the survival function value). (The alternative approach of multiplying the mortality probability by the number of live eggs introduces a number of numerical difficulties when the number of live eggs is small.) The number of eggs dying over the whole time step is calculated as the number of eggs that die each day multiplied by the parameter *timestep-scale* (it only applies to low and high temperature as well as dewatering mortality sources, since superimposition mortality only occurs once per time step and scouring results in mortality of all eggs in the redd).

The separate redd mortality sources are executed sequentially: the eggs killed by one source are subtracted from the number alive before the next source is processed. The order in which redd survival functions are evaluated is as follows:

1.7.6.1. Low temperature:

The daily fraction of eggs surviving low temperatures is modeled as an increasing logistic function of temperature.

1.7.6.2. High temperature:

The fraction of eggs surviving high temperatures is modeled as a decreasing logistic function of temperature.

1.7.6.3. Scouring and deposition:

Scouring and deposition mortality results from high flows disturbing the gravel containing a redd. The model assumes that the probability of a redd being destroyed is equal to the proportion of the stream reach scouring or filling to depths greater than the value of the fish parameter *mortReddScourDepth* (cm). Consequently, the probability of a redd not being destroyed (*scourSurvival*) is equal to the proportion of the stream scouring or filling to a depth less than the value of *mortReddScourDepth*. This scour survival probability is estimated from the exponential distribution model of Haschenburger (1999); the proportion of the stream scouring to less than a given depth is the integral of the exponential distribution between zero and the depth:

*scourSurvival* =1 - e^-(^*^scourParam^* ^×^ *^mortReddScourDepth^*^)^ (46)

The value of *scourSurvival* is set to 1.0 if (*scourParam* × *mortReddScourDepth*) is greater than 100. The value of *scourParam* was modeled by Haschenburger (1999) empirically:

*scourParam* = 3.33 × e^-1.52 (shearStress / 0.045)^ (47)

where *shearStress* is the peak Shields stress (measured at a reach scale) occurring during the high-flow event. Shields stress is a dimensionless indicator of scour potential often used in modeling sediment transport, described in the sediment transport literature. Shields stress increases with flow, a relationship represented in the model by the equation:

*shearStress* = *habShearParamA* × (*flow)^habShearParamB^* (48)

where *habShearParamA* (s m^-3^) and *habShearParamB* (unitless) are habitat reach parameters. Since *scourSurvival* is 1.0 when (*scourParam* × *mortReddScourDepth*) is greater than 100, this allows users to effectively turn scouring and deposition mortality off by using a very large value of *mortReddScourDepth*, e.g., 10,000 cm.

1.7.6.4. Dewatering:

Dewatering mortality occurs when flow decreases until a redd is no longer submerged. The dewatering survival function is simply that if depth is zero then the daily fraction of eggs surviving is equal to the fish parameter *mortReddDewaterSurv*.

1.7.6.5. Superimposition:

Superimposition redd mortality can occur when a new redd is laid over an existing one. Importantly, superimposition only occurs when the redds are laid in gravel. Otherwise, it is assumed that they cannot be disturbed by another spawner. Therefore, in the event that *cellFracGravel* is zero, there is no risk of superimposition. Otherwise, superimposition redd mortality is modelled as a function of the area disturbed in creating the new redd and the area of spawning gravel available. The following steps are used for each redd, for each time step:

1. Determining if one or more new redds were created in the same cell on the current time step. If not, then superimposition survival is 1.0.

2. If one or more redds were created in the same cell, the probability of each new redd causing superimposition (*reddSuperImpRisk*, unitless) is equal to the area of a redd (*reddSize*, cm^2^) divided by the area of spawning gravel in the redd.

*reddSuperImpRisk* = *reddSize* / (*cellArea* × *cellFracGravel*) (49)

3. A random number is drawn from a uniform distribution between zero and one; if it is less than *reddSuperImpRisk*, then superimposition mortality occurs.

4. If superimposition mortality occurs, then the fraction of eggs surviving is the value of another random number drawn from a uniform distribution between zero and one.

5. Steps 2-4 are executed once for each new redd placed in the cell on the current time-step.

*1.7.7. Redd Development*:

To predict the timing of emergence, the developmental status of a redd’s eggs is updated daily. We used the fractional development approach of Van Winkle et al. (1996) which is based on accumulated degree-days. Model redds accumulate the fractional development that occurs each day (*reddDailyDevel*), a function of temperature. This means the redd has a state variable *fracDeveloped* that starts at zero when the redd is created and is increased each day by the value of *reddDailyDevel*. When *fracDeveloped* reaches 1.0, then the eggs are ready to emerge. The daily value of *reddDailyDevel* is determined using this second-order polynomial equation:

*reddDailyDevel* = *reddDevelParamA* + (*reddDevelParamB* × *temp*) (50)

+ (*reddDevelParamC* × *temp*^2^)

The fractional development that occurs over the whole time step is then calculated as the daily development multiplied by the parameter *timestep-scale*.

*1.7.8. Emergence from the redds and Transmission of traits*:

1.7.8.1. Emergence:

“Emergence” is the conversion of each surviving egg into a new trout agent. Emergence begins on the day when *fracDeveloped* reaches 1.0, and then the new fish emerge over a period of several days. As a simple way to spread emergence over several days, the emergence model assumes that 10% of the redd’s eggs emerge on the first day of emergence; 20% of the redd’s remaining eggs emerge on the next day; 30% of the remaining eggs emerge on the third day; etc, until 100% of remaining eggs emerge. The time at which all eggs have emerged depends on the time step defined by the user through the parameter *timestep-scale*. As emergence proceeds, the eggs remaining in a redd remain susceptible to egg mortality.

1.7.8.2. Transmission of heritable traits:

For modelling the transmission of heritable traits, we followed the approach of Vincenzi et al. (2012). Only length at emergence and the size threshold value for maturation are considered genetically coded and heritable. We additionally included a neutral trait (not affecting the fitness of individuals) to assess whether potential changes along time in the genotypic values of heritable traits are actually due to directional selection and not by genetic drift. We assume that each egg is fertilized by just one male spawner. Therefore, each new trout emerging from the redd inherits its traits from a father randomly assigned from the *number-males* males contributing to the redd (see Section 1.7.2.2).

As commonly modelled (Lynch and Walsh 1998), the phenotype z of an individual *i*, *z*_i_, is defined in our model as the sum of its genotypic (also called breeding) value *a*_i_ (representing additive genetic variance) randomly drawn from a normal distribution N($\mu_{G}$, $\sigma_{G}^{2}$), and a statistically independent random environmental effect from N($\mu_{E}$, $\sigma_{E}^{2}$):

*z*_i_ = *a*_i_ + *e*_i_ (51)

where the narrow-sense heritability *h*^2^ = $\sigma_{G}^{2}$ / $\sigma_{Z}^{2}$ indicates how much of the phenotypic variance $\sigma_{Z}^{2}$ present in the population is explained by the additive genetic variance $\sigma_{G}^{2}$.

In our model, a genotypic value *genTraitZ* of the heritable traits is set at initialization of the individuals following a normal distribution around the mean phenotypic value at the population level (*fishTraitZMean*; input parameter) and with an additive genetic variance computed as:

*additivevarTraitZ* = $h_{TraitZ}^{2}$ × *fishTraitZVar* (52)

where *fishTraitZVar* is the phenotypic variance of the trait at the population level and $h_{TraitZ}^{2}$ is the narrow-sense heritability of the trait. Both *fishTraitZVar* and $h_{TraitZ}^{2}$ are input parameters to the model.

The phenotypic expression of the trait (*fishTraitZ*) for each individual at initialization is then calculated as *genTraitZ* plus the environmental effect drawn from a normal distribution with mean 0 and an environmental variance $\sigma_{E}^{2}$ equal to (1 - $h_{TraitZ}^{2}$) × *fishTraitZVar*. The environmental variance is maintained constant along the whole simulation.

Inheritance rules are based on the infinitesimal model of quantitative genetics theory. Each offspring's *genTraitZ* value for a trait *z* under selection is drawn from a normal distribution centered on the arithmetic mean of the two parental values, while the variance of this distribution is equal to half the total additive genetic variance for the trait at the population level (i.e., the within-family additive variance remains constant).

In an idealized population with no input of new variation from mutation or migration, the additive variance generated from the initial variation in the base population eventually declines. Ultimately, a selection limit or plateau is reached, and as the genetic variation in the base population becomes exhausted, the effects of new mutations become increasingly important for continued response (Johnson and Barton 2005). In our model, the user has the option to choose whether the total additive genetic variance fixed at initialization remains constant across generations or it changes otherwise, being then computed as the variance of the breeding genotypic values. The inheritance model is a modified version of the inheritance model of the infinitesimal model of quantitative genetics theory, adapted to account for new input of variation from mutation. Offspring then inherit the trait *genTraitZ* from a normal distribution centered on the arithmetic mean of the two parental values and with the variance $\sigma_{G,off}^{2}$ of the distribution equal to half the mean of population additive genetic variance (*additivevarTraitZ*) plus the mutational variance $\sigma_{m}^{2}$ multiplied by a factor *M* defining the amplitude of mutation:

$\sigma_{G,off}^{2}=\frac{1}{2}(additivevarTraitZ+M\sigma_{m}^{2})$ (53)

In our model, the mutational variance $\sigma_{m}^{2}$ (variance introduced by mutation per generation) at the population level is computed as (*mutationalVarParam* × $\sigma_{E}^{2}$), where *mutationalVarParam* is in the order of 10^-3^ to 10^-2^, as suggested by reviews of empirical data (Lynch and Walsh 1998, Johnson and Barton 2005). Variation from mutation can be turned off by the user by setting the amplitude of mutation factor *M* to 0.

The environmental *e*_i_ component of the trait is drawn from a normal distribution of mean 0 and variance equal to the environmental variance $\sigma_{E}^{2}$fixed at initialization. Offspring phenotypes are then formulated as *fishTraitZ* = *genTraitZ* + *e*_i_.

The model allows the possibility of defining different maturation thresholds for males and females. Therefore, the way this trait is transmitted is slightly different from the other two heritable traits. We used a standard transformation for this purpose. The parental genotypic values of the trait are standard transformed as follows:

(54)

$StdParentgenSpawnMinLength =\frac{ParentgenSpawnMinLength - SexPopMeangenSpawnMinLength}{\sqrt{\frac{1}{2}(additivevarSpawnMinLength+M\sigma_{m}^{2})}}$ ,

where *StdParentgenSpawnMinLength* is the standard transformed of the genotypic value of either the father or the mother (*ParentgenSpawnMinLength*), *SexPopMeanSpawnMinLength* is the mean genotypic value of either males or females at the population level (depending on the sex of the new trout and equal to *SpawnMinLengthMean* if additive variance is fixed across generations or computed as the mean genotypic value of either male or female breeder population otherwise), *additivevarSpawnMinLength* is the additive genetic variance for the trait and $M\sigma_{m}^{2}$ represents the mutational variance. The two parental standardized values are then averaged (*stdMean*) and a random number (*randStd*) is drawn from a normal distribution N(*stdMean*, 1). Finally, the genotypic value of the maturity threshold for the new trout is calculated by transforming back as follows:

*genSpawnMinLength* = *SexPopMeanSpawnMinLength* (55)

+ *randStd* × $\sqrt{\frac{1}{2}(additivevarSpawnMinLength+M\sigma_{m}^{2})}$

Both the genotypic value and the environmental component of the heritable traits are truncated at 4 standard deviations from the centers of the normal distributions from which they are drawn.

*1.7.9. Trout Ageing*:

Trout update every time step their state variable *age*, which track the number of days since a trout was born. Trout update their *age-class* state variable the Julian date where they were born. When new trout are born, then all trout from the older cohorts update their age-class to avoid that some fish could have the same age-class while belonging to different cohorts. The same action happens likewise when the first fish of older cohorts updates its age-class and new recruits have not been born yet.

**Appendix S2:**

**Fishing module description**

The fundamental assumption of the fishing model formulation is that the risk to an individual fish of being hooked by anglers is a function of fishing pressure and not directly a function of trout abundance. The model assumes anglers fish a constant fraction of the catchable population (i.e., the exploitation rate is fixed), so more fish are harvested when the harvestable stock abundance is higher. The risk of a trout being hooked decreases sharply at low body sizes (Figure S2-1) and the model assumes that an individual trout can be caught more than once in a day. Trout can be only angled during the angling season, defined by the fishery-specific parameters *startAnglingSeason* and *endAnglingSeason*. Angling mortality risk does not vary with habitat, so simulated trout cannot avoid it via their habitat selection behaviour. The fishing model is executed as follows:

1. The daily capture rate is calculated from the fishing pressure and the trout’s length. Fishing pressure (variable *anglePressure*) is evaluated as angler hours per day per km of stream, and is determined by the exploitation rate (*ExpR*) determines the fishing pressure as follows:

*anglePressure* [angler-hr km^-1^ day^-1^] = (((*ExpR* [trout caught per trout harvestable] / 100) × *harvestableStock* [trout harvestable] × *anglingEfficiency* [angler-hr trout caught^-1^]) / (*reachLength* [cm] × 10^-5^ [km cm^-1^] × *seasonLength* [day]))

Capture rate is represented as the average number of times a fish is hooked per day. This capture rate is assumed to be a linear function of fishing pressure, with the proportionality constant being the parameter *mortFishAngleSuccess*. This parameter represents fishing success as the fraction of catchable fish hooked per angler hour. Capture rate is also assumed to be a logistic function of trout size. The capture probability function is:

*captureRate* [trout caught per trout catchable per day] = *mortFishAngleSuccess* [trout caught per trout catchable per angler-hr] × *anglePressure* [angler-hr km^-1^ day^-1^] × *reachLength* [cm] × 10^-5^ [km cm^-1^] × logistic(*fishLength*)

where the logistic function of fish length (Figure S2-1) is defined by two trout parameters *mortFishAngleL1* and *mortFishAngleL9*.

2. The number of times a trout is hooked during a time-step (variable *timesHooked*) is drawn from a Poisson distribution parameterized with the capture rate (average captures per day). If *timesHooked* is zero, the survival probability is 1.0 for angling and hooking mortality.

3. If *timesHooked* is one or more, the model first determines whether it is legal to keep the trout according to a harvest slot limit: it is legal to keep trout that have length greater than the value of the parameter *MinLL* (cm) and lower than the value of *MaxLL* (cm). Steps 4 and 5 are subsequently performed once for each time the trout is hooked:

4. If the trout is of legal size to keep, a random draw is applied to the parameter *mortFishAngleFracKeptLegal* to determine whether the trout is kept. If the trout is not of legal size, a random draw and the parameter *mortFishAngleFracKeptIllegal* determine whether the trout is kept. If the trout is kept, the survival probability is 0.0. Hooked fish that are kept by anglers are considered dead by “angling”. Fish that die of angling mortality are not subject to hooking mortality.

5. If the trout is released (not kept), the survival probability for angling is 1.0, but the fish is then subject to the hooking mortality source. The probability of surviving hooking mortality is defined by the parameter *mortFishAngleHookSurvRate*. Trout that do not survive are considered dead by “hooking”.


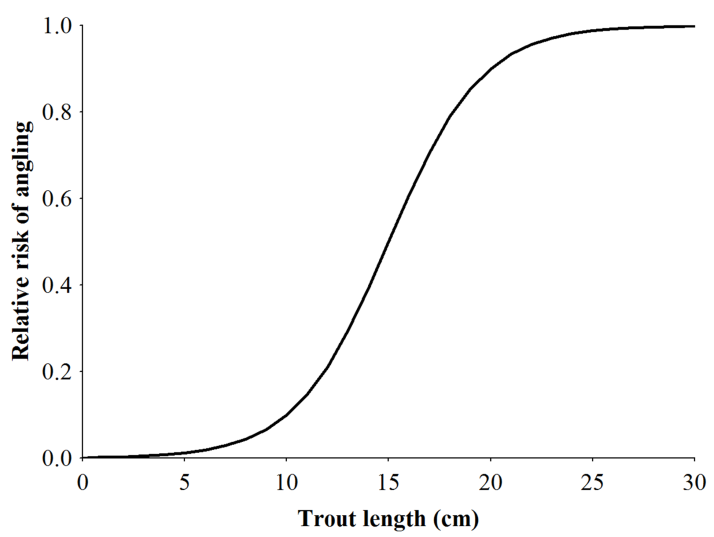


**Figure S2-1.** Function describing the size-dependency on the relative risk of being hooked for trout.

**Appendix S3:**

**Model parameterization**

We parameterized inSTREAM-Gen for a resident brown trout population in the River Belagua, in the Aragón River basin (northern Spain). The study river corresponds to the Mediterranean medium-sized headwater mountain stream reach type described in Ayllón et al. (2010), and the length of the simulated reach is 305 meters. We selected this river because it is free from any human pressure other than fishing, and the fishing pressure is low (exploitation rate ­–percentage of trout legally harvested from the harvestable stock– below 8%), and therefore represents a good test case to show how increasing exploitation intensity may alter the eco-evolutionary trajectory in an almost non-fished population. Model parameterization is thoroughly described in Ayllón et al. (2016). We performed pattern-oriented calibration of six parameters to reproduce density, biomass and size-at-age annual patterns recorded in the field in summer over 1993-2004 (summarized in Table S3-1), which represents the population’s size- and age-structure at the beginning of the simulations. Under this natural population structure, the ratio of adults to juveniles biomass is 2.27. The genotypic value of the minimum length for spawning is the same for males and females (16.50 ± 1.22 cm), while the genotypic value of length at emergence is 2.30 ± 0.15 cm. The narrow-sense heritability *h^2^* of the quantitative genetic parameters was set to 0.2 and 0.18, respectively.

**Table S3-1.** Density, biomass and mean size-at-age averaged (± standard deviation) for the 1993-2004 period, both total and broken out by age-classes. These population patterns were used for model calibration, and therefore represent the population structure at the beginning of the simulations.

| **Pattern** | **Total** | **Age-0** | **Age-1** | **Age-2** | **Age-3** | **>Age-3** |
| --- | --- | --- | --- | --- | --- | --- |
| *Density (trout/ha)* | 3053.9 ± 1448.6 | 1418.9 ± 1284.4 | 1040.2 ± 669.7 | 476.1 ± 310.8 | 94.8 ± 62.1 | 30.6 ± 24.2 |
| *Biomass (kg/ha)* | 78.5 ± 28.8 | 3.6 ± 3.5 | 23.3 ± 14.8 | 31.6 ± 20.3 | 13.0 ± 7.7 | 16.6 ± 3.3 |
| *Length-at-age (cm)* | - | 6.0 ± 0.9 | 12.4 ± 1.5 | 17.8 ± 1.4 | 22.7 ± 1.6 | 29.1 ± 2.7 |
| *Weight-at-age (g)* | - | 2.5 ± 1.1 | 22.4 ± 8.0 | 66.2 ± 15.7 | 136.7 ± 29.5 | 294.2 ± 90.6 |

The fishing module was parameterized with site-specific data provided in Almodóvar et al. (2006) (Table S3-2). These data are based on creel-surveys conducted over the period 1994-2002 by the Wildlife Regional Service of Navarra. Values used to parameterize the logistic function relating fish length and capture rate, and to define the probability of surviving hooking mortality were derived from Railsback et al. (2006), as they were not available for the modelled population.

The rate of hooking mortality selected (20%) was relatively high but within the ranges observed for th species and close to the average value reported for salmonids typically fished in European fisheries (15.9±1.4%; see meta-analysis by Hühn and Arlinghaus 2011). It is a compromise between the average value (12.1±6.7%) reported for brown trout fished with live bait (the one typically used in the modelled fishery), and the average value (27%) reported for salmonids with the same bait type (Hühn and Arlinghaus 2011). These figures include mainly results from short-term experiments (≤ 3-day observations); the long-term (14-day observations) average hooking mortality rate for brown trout fished with live bait reported by Hulbert and Engstrom-Heg (1980) increases to 13.4±5.7%, with reported values up to 23%.

The values of the parameters defining the probability of anglers keeping trout of either legal or illegal size were obtained via calibration to match patterns of annual harvest and exploitation rate observed historically in the modelled river reach (available in Almodóvar et al. 2006). The values of three parameters vary according to the simulated scenarios described in the main manuscript.

**Table S3-2.** Parameter values used in the fishing module (SA - varying according to simulated scenarios, * site-specific values from Almodóvar et al. 2006, ** from Railsback et al. 2006, *** from Hühn and Arlinghaus 2011, ++ calibrated).

| **Parameter** | **Definition (units)** | **Value** |
| --- | --- | --- |
| *startAnglingSeason* | Start of the angling season (Julian date) | 91* |
| *endAnglingSeason* | End of the angling season (Julian date) | 274* |
| *anglePressure* | Fishing pressure (angler-h km^-1^ d^-1^) | SA |
| *MinLL* | Lower bound of the length range in which fish are legal to keep (cm) | SA |
| *MaxLL* | Upper bound of the length range in which fish are legal to keep (cm) | SA |
| *mortFishAngleSuccess* | Multiplier to determine capture probability from fishing pressure (angler-h)^-1^ | 0.02 * |
| *anglingEfficiency* | Number of angler hours necessary to catch and keep a trout (angler-h) | 3.7 * |
| *mortFishAngleL9* | Length at which hooking risk is 90% of maximum (cm) | 20 ** |
| *mortFishAngleL1* | Length at which hooking risk is 10% of maximum (cm) | 10 ** |
| *mortFishAngleFracKeptLegal* | Probability of fish of legal length being kept by anglers (unitless) | 0.40 ++ |
| *mortFishAngleFracKeptIllegal* | Probability of fish not of legal length being kept by anglers (unitless) | 0.05 ++ |
| *mortFishAngleHook-SurvRate* | Survival probability for released trout (or trout that shake the hook) (unitless) | 0.8 *** |

***Literature cited:***

Almodóvar, A., G.G. Nicola, B. Elvira, D. Ayllón, S. Leal, and I. Parra. 2006. Analysis of population dynamics and angling impacts on the brown trout populations of Navarre. Carrying capacity modelling for brown trout population conservation and management. Study of the genetic variability of brown trout in Navarre. (*In Spanish*). Report. Government of Navarre, Spain.

Ayllón, D., A. Almodóvar, G. G. Nicola, and B. Elvira. 2010. Ontogenetic and spatial variations in brown trout habitat selection. Ecology of Freshwater Fish **19**:420-432.

Ayllón, D., S. F. Railsback, S. Vincenzi, J. Groeneveld, A. Almodóvar, and V. Grimm. 2016. InSTREAM-Gen: Modelling eco-evolutionary dynamics of trout populations under anthropogenic environmental change. Ecological Modelling **326**:36-53.

Hühn, D., and R. Arlinghaus. 2011. Determinants of hooking mortality in freshwater recreational fisheries: a quantitative meta-analysis. American Fisheries Society Symposium 75:141-170.

Hulbert, P.J., and R. Engstrom-Heg. 1980. Hooking mortality of worm-caught hatchery brown trout. New York Fish and Game Journal **27**:1-10.

Railsback, S. F., J.W. Hayes, and K.E. LaGory. 2006. Simulation analysis of within-day flow fluctuations on trout below Flaming Gorge Dam. Argonne National Laboratory, ANL/EVS/TM/06-01, Argonne, Illinois, USA.

**Appendix S4:**

**Variability of model outputs between replicates**

**Table S4-1.** Coefficient of variation (%) between six replicates of the mean value over the last 15 simulated years (2086-2100) of model outputs under fishing scenarios involving the combination of four levels of exploitation rate (20, 35, 50 and 65%) and three levels of the minimum-length limit (17, 19 and 21 cm), while the maximum-length limit is fixed to 100cm.

| **Exploitation rate:** | **20%** | | | **35%** | | | **50%** | | | **65%** | | |
| --- | --- | --- | --- | --- | --- | --- | --- | --- | --- | --- | --- | --- |
| **Min-length limit :** | **17** | **19** | **21** | **17** | **19** | **21** | **17** | **19** | **21** | **17** | **19** | **21** |
| **Model outputs** |  |  |  |  |  |  |  |  |  |  |  |  |
| Density total | 0.6 | 0.1 | 0.5 | 0.7 | 1.1 | 0.7 | 1.1 | 1.2 | 1.1 | 1.6 | 1.6 | 1.4 |
| Density age-0 | 0.8 | 0.4 | 0.6 | 1.2 | 1.3 | 0.7 | 0.8 | 1.3 | 1.5 | 1.6 | 1.6 | 1.7 |
| Density age-1 | 0.8 | 1.3 | 0.7 | 2.2 | 0.8 | 2.5 | 1.6 | 0.1 | 0.7 | 2.5 | 1.2 | 1.6 |
| Density age-2 | 1.7 | 1.3 | 2.8 | 1.8 | 2.4 | 2.2 | 7.3 | 1.7 | 2.8 | 3.7 | 5.8 | 2.6 |
| Density age-3Plus | 5.7 | 7.0 | 7.1 | 3.6 | 6.9 | 3.3 | 21.9 | 16.3 | 7.3 | 22.0 | 16.9 | 6.2 |
| Weight age-0 | 1.0 | 0.2 | 1.0 | 0.3 | 1.2 | 1.3 | 1.9 | 0.9 | 1.0 | 1.5 | 1.3 | 1.2 |
| Weight age-1 | 3.1 | 0.7 | 1.1 | 2.0 | 1.6 | 3.4 | 1.9 | 0.6 | 1.4 | 1.1 | 1.4 | 1.1 |
| Weight age-2 | 3.7 | 2.1 | 1.9 | 1.5 | 1.5 | 2.0 | 1.8 | 1.7 | 2.7 | 3.9 | 4.6 | 1.5 |
| Weight age-3Plus | 6.5 | 2.0 | 4.2 | 6.1 | 1.6 | 1.0 | 17.3 | 5.2 | 2.2 | 24.4 | 7.4 | 2.6 |
| Biomass total | 0.8 | 0.6 | 2.3 | 0.2 | 0.9 | 1.0 | 1.1 | 1.4 | 1.0 | 0.5 | 2.1 | 1.6 |
| Biomass age-0 | 1.1 | 0.7 | 0.9 | 1.1 | 0.5 | 1.4 | 1.0 | 0.8 | 0.6 | 0.4 | 0.6 | 1.1 |
| Biomass age-1 | 2.6 | 1.2 | 1.2 | 1.6 | 2.4 | 1.5 | 0.8 | 0.5 | 0.5 | 1.6 | 2.5 | 1.3 |
| Biomass age-2 | 2.0 | 3.1 | 2.8 | 3.1 | 2.9 | 1.6 | 5.4 | 0.5 | 3.1 | 6.6 | 3.4 | 1.7 |
| Biomass age-3Plus | 11.6 | 5.9 | 9.4 | 9.7 | 6.1 | 3.9 | 26.4 | 13.4 | 8.4 | 30.2 | 17.7 | 5.7 |
| Ratio adults to juveniles | 5.5 | 0.1 | 7.3 | 0.1 | 0.6 | 2.9 | 9.2 | 3.7 | 0.0 | 0.8 | 4.4 | 1.7 |
| Density spawners | 11.2 | 3.8 | 4.0 | 5.4 | 2.4 | 6.2 | 1.6 | 4.7 | 5.9 | 3.6 | 8.6 | 2.4 |
| Number eggs | 3.5 | 7.8 | 4.9 | 2.5 | 3.4 | 4.8 | 1.8 | 7.2 | 7.2 | 5.5 | 7.3 | 6.4 |
| Spawning Age Male | 2.4 | 1.2 | 0.7 | 2.9 | 0.8 | 0.5 | 1.1 | 2.9 | 2.0 | 2.7 | 2.9 | 1.7 |
| Spawning Age Female | 3.3 | 2.0 | 3.3 | 1.0 | 1.8 | 1.8 | 2.4 | 1.1 | 0.7 | 1.3 | 0.4 | 1.6 |
| Gen Min Spawn Length Male | 2.5 | 1.3 | 0.5 | 1.6 | 1.1 | 1.8 | 0.5 | 0.5 | 1.7 | 0.6 | 1.7 | 0.8 |
| Gen Min Spawn Length Fem | 2.6 | 1.4 | 0.5 | 1.5 | 1.0 | 1.7 | 0.5 | 0.4 | 1.7 | 0.5 | 1.7 | 0.9 |
| Gen Emergence Length | 1.1 | 1.0 | 0.7 | 1.4 | 0.8 | 1.0 | 1.4 | 1.2 | 1.5 | 1.4 | 0.7 | 1.9 |
| Hooked dead fish (trout/ha) | 0.4 | 2.7 | 3.7 | 2.3 | 2.0 | 4.5 | 2.3 | 0.6 | 4.4 | 0.7 | 6.5 | 2.1 |

**Appendix S5:**

**Trends analysis**

***Data analysis***

We evaluated annual trends of model outputs using the Mann-Kendall trend test (Mann 1945, Kendall 1975), as modified by Yue et al. (2002) to account for serial autocorrelation. This non-parametric rank-based test determines whether there is an upward or downward trend over time in the variable, the null hypothesis being no trend (the data is independent and randomly ordered). An upward (downward) trend thus means that the variable consistently increases (decreases) through time, but the trend may or may not be linear. This test is widely used because it is robust to outliers and nonlinear trends, does not require the data to be normally distributed, and has low sensitivity to abrupt breaks due to non-homogeneous time series (Gilbert 1987, Esterby 1996). We used the *zyp* v0.10-1 R package (Bronaugh and Werner 2015) to estimate the Kendall's tau statistic and its probability, which indicates whether the trend is significant or not. In addition, we calculated the Sen's slope, i.e. the median linear slope joining all pairs of observations, expressed as percent of the mean quantity per unit time.

***Results***

Figure S5-1 shows the ratio of life-history and demographic outputs under fishing scenarios to the baseline scenario over time. Figure S5-2 shows the results from the Mann-Kendall test for all model outputs under tested fishing scenarios. Figure S5-3 shows the percentage change after 25 years of simulation of model outputs from the baseline scenario of no angling.


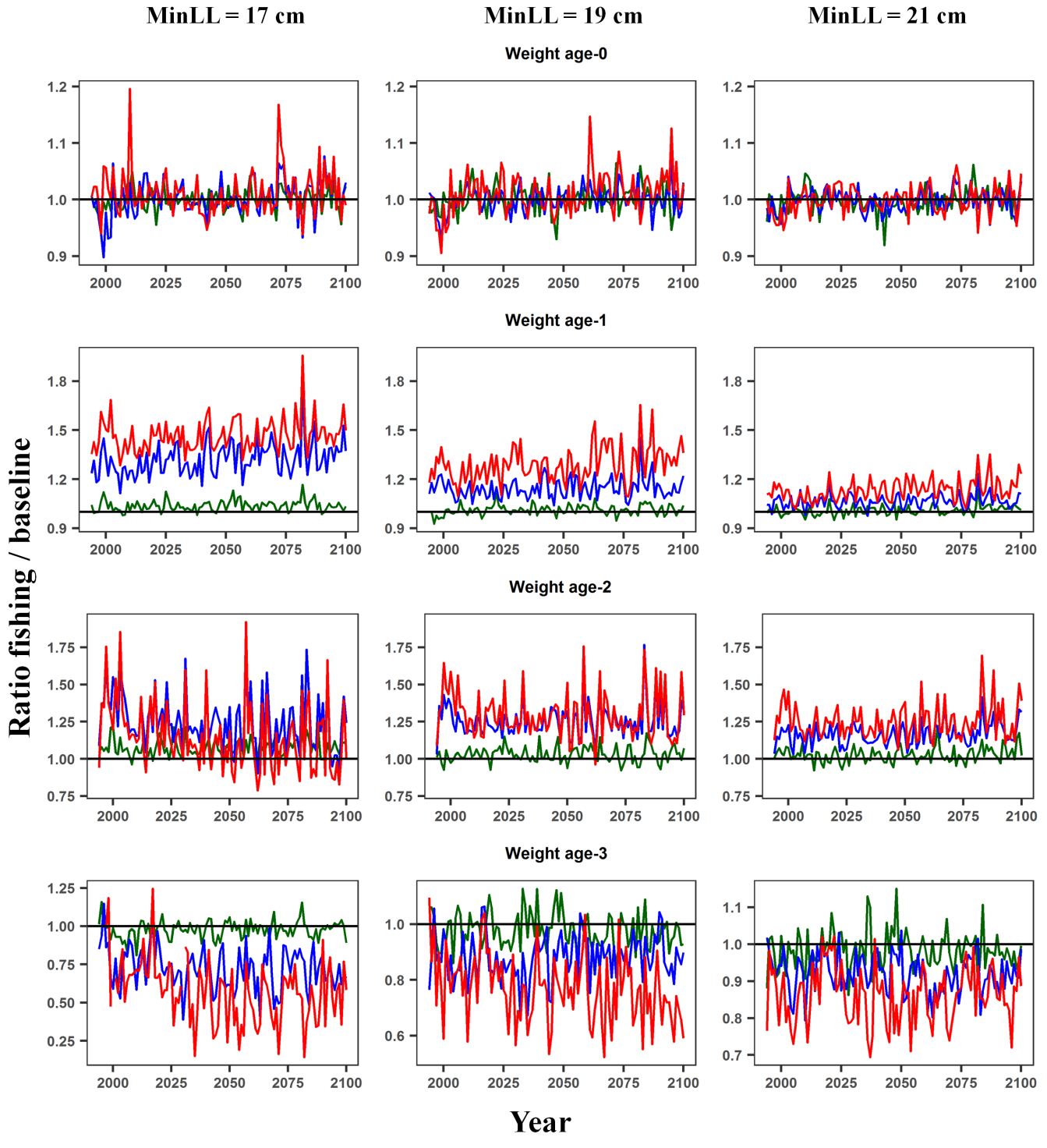


**Figure S5-1.** Ratio of life-history (mean individual weight) and demographic (density and biomass) model outputs for four age classes under different angling scenarios to the baseline scenario (no angling and hooking mortality simulated) over time. Represented angling scenarios are combinations of three levels of minimum-length limit (17, 19 and 21 cm) and exploitation rate (5, 35 and 65%, plotted as green, blue and red lines, respectively). The maximum-length limit was set to 100 cm (i.e., no slot-length limits, but minimum-length limits regulation applied).


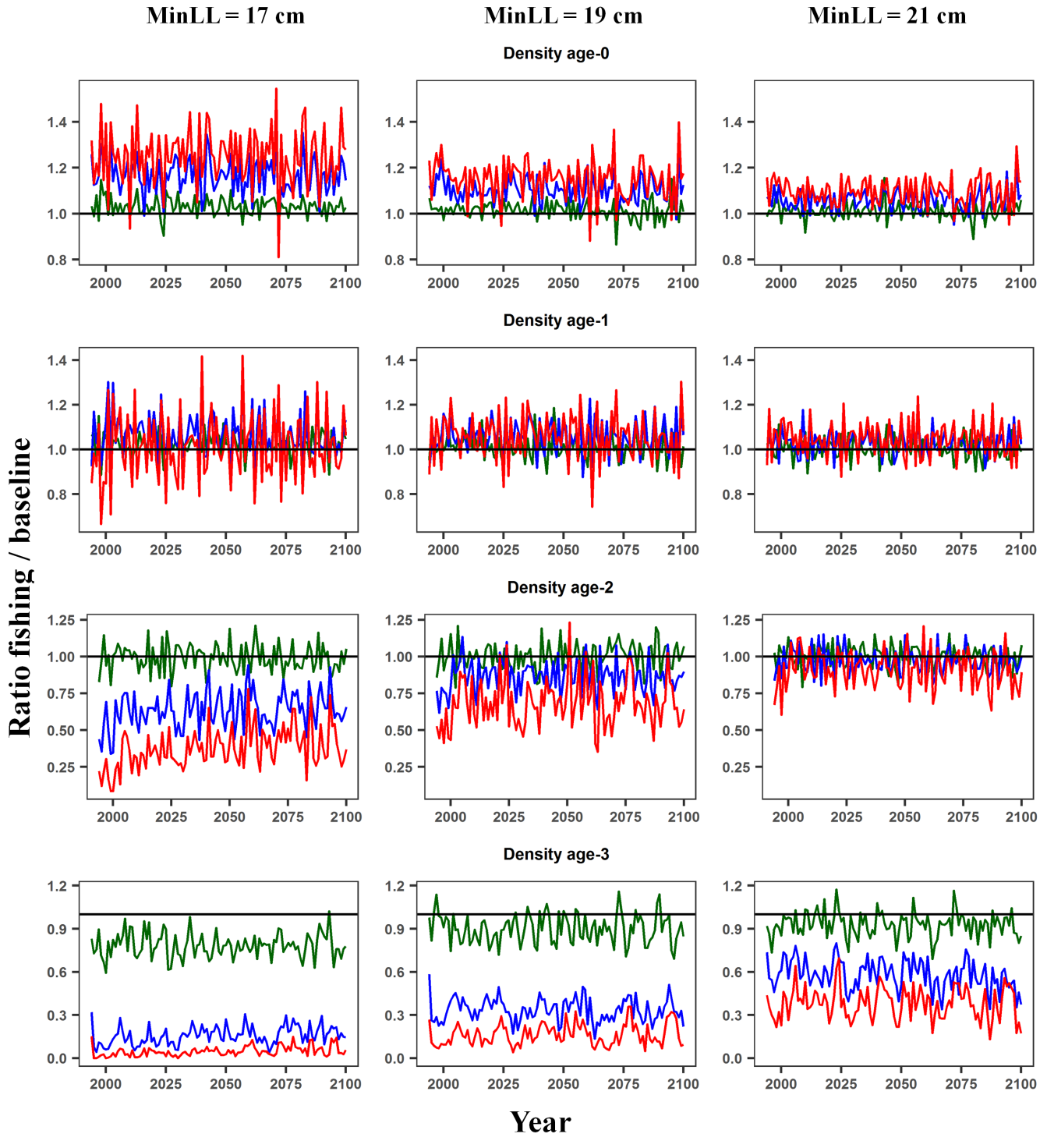


**Figure S5-1.** Continuation.


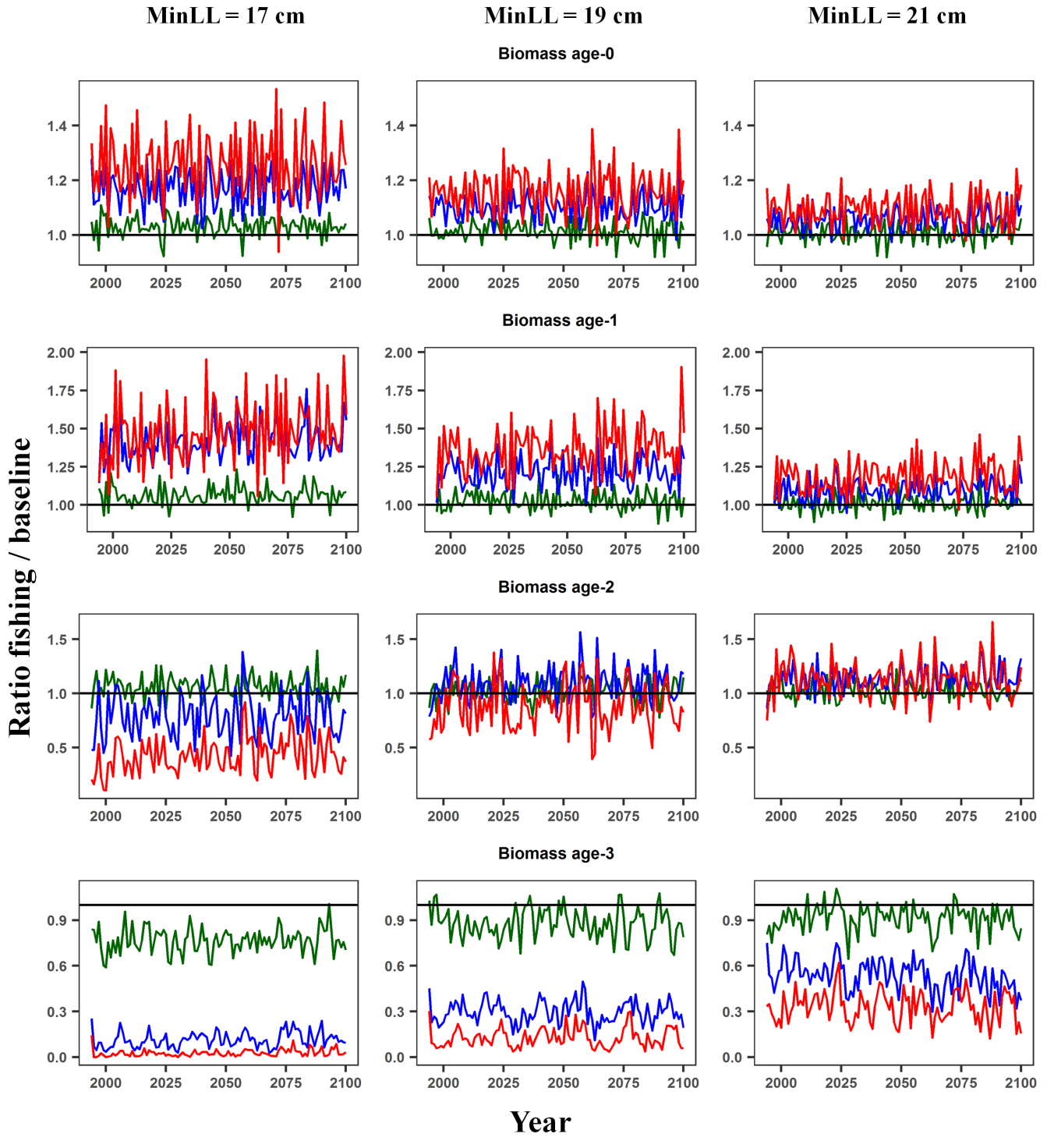


**Figure S5-1.** Continuation.


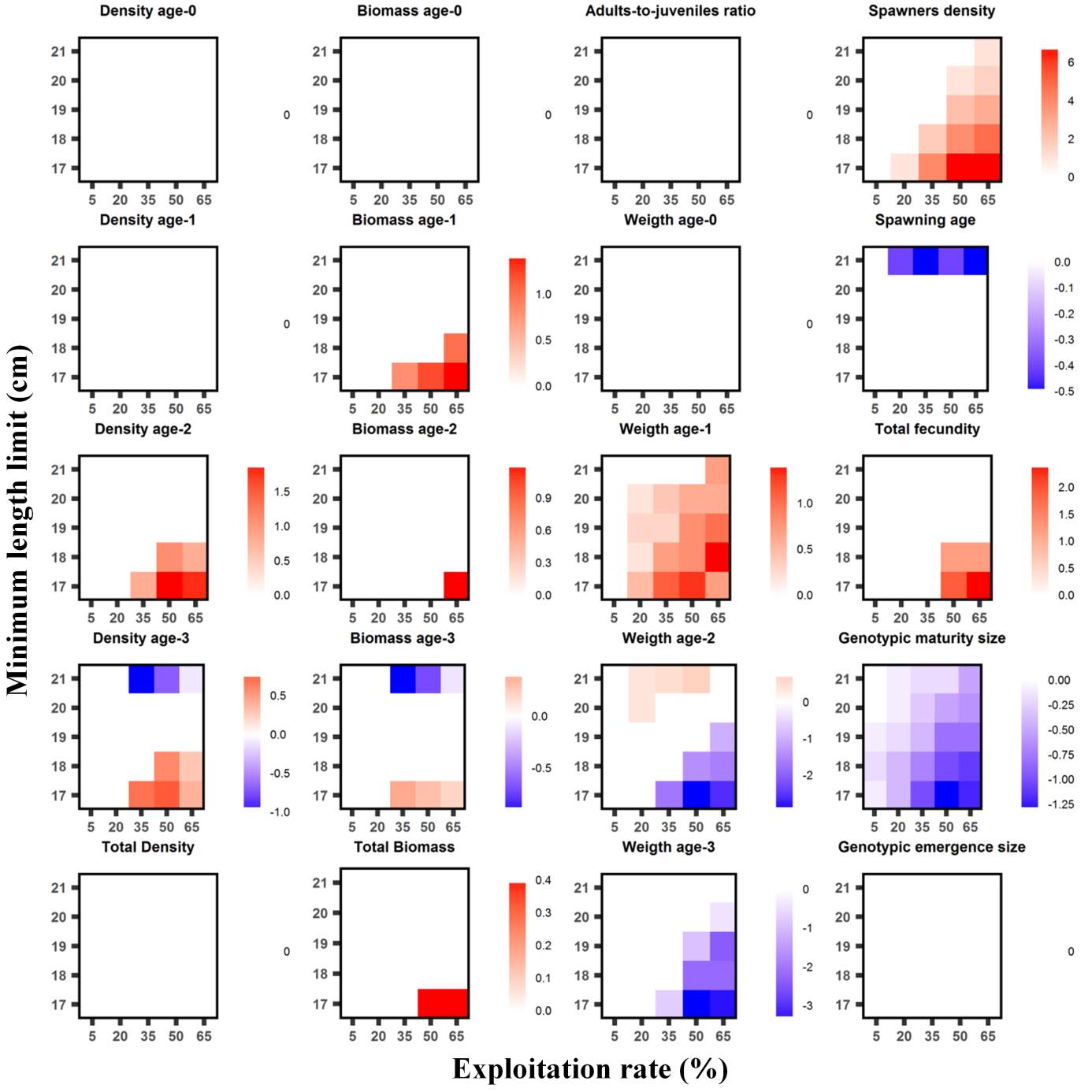


**Figure S5-2.** Trends of model outputs over the 1994-2100 time period for the angling scenarios resulting from the combination of tested exploitation rate and minimum-length limit levels, while the maximum-length limit is set to 100 cm (i.e., no slot-length limits, only minimum-length limit). Tested variables were represented as the ratio of their value under the angling scenario to their value under the baseline scenario. The baseline refers to the scenario in which no angling and hooking mortality were simulated. For each graph, a white colour indicates that there were no significant increasing or decreasing trends of magnitude over time (*P* <0.05). Blue/red shades indicate significant rates of change over time, measured as the Sen's slope (% / decade).

**Fig. S5-3.** Effect of exploitation rate and minimum-length limit on population eco-evolutionary outputs after 25 years of simulation for maximum-length limit of 100 cm (i.e., no slot-length limits, only minimum-length limit). For each graph, a white colour indicates that the simulation results under the angling scenario are not significantly different (pairwise t-tests, *P* <0.05) from the baseline scenario of no angling. Blue/red shades indicate the strength of decrease/ increase of the angling scenario compared to the baseline. Colour scales on the right of each graph indicate the ranges of significantly different mean values over the first 25 simulated years expressed as the percentage change: [(mean scenario - mean baseline)/mean baseline] x 100. Total density, density of age-1 trout, weight of age-0 trout, genotypic length-at-emergence, and neutral trait values of spawners did not significantly differ from the baseline scenario under any angling scenario so are not graphed.

***Literature cited***

Bronaugh, D., and A. Werner. 2015. zyp: Zhang + Yue-Pilon trends package. R Package Version 0.10-1.

Esterby, S.R. 1996. Review of methods for the detection and estimation of trends with emphasis on water quality applications. Hydrological Processes 10:127-149.

Gilbert, R.O. 1987 . Statistical Methods for Environmental Pollution Monitoring. Wiley, New York.

Kendall, M. 1975. Multivariate Analysis. Charles Griffin & Company, London.

Mann, H.B. 1945. Nonparametric tests against trend. Econometrica 13:245-259.

Yue, S., P. Pilon, B. Phinney and G. Cavadias. 2002. The influence of autocorrelation on the ability to detect trend in hydrological series. Hydrological Processes 16:1807-1829.

**Appendix S6:**

**Comparison of simulations with vs. without evolution**

For this analysis, we simulated the effects of intensive fishing (exploitation rates ranging from 35 to 65% ) together with the least restrictive harvest regulation (MinLL= 17 cm, MaxLL=100 cm) under a scenario in which evolution was not allowed and compared them to the results from the simulations performed with evolution being modelled. The rest of simulation conditions were identical across scenarios with and without evolution. The purpose of this analysis was to test the actual contribution of evolutionary responses to buffering the negative impacts of harvesting.

The numbers and biomass of age-2 and older trout and thus the ratio of adults to juveniles biomass decreased under the scenarios without micro-evolution compared to those in which genetic traits were allowed to evolve (Table S6-1). Likewise, the number of spawners and total fecundity was reduced when simulations did not account for micro-evolutionary processes. The beneficial effect of fishing-induced evolution increased with increasing exploitation rate.

**Table S6-1.** Differences in the population outputs endpoints between simulations in which micro-evolution is not allowed and simulations with evolution modelled under three different levels of exploitation rate (35, 50 and 65%), with fixed minimum- (17 cm) and maximum-length (100 cm) limits. Differences in the mean values over the last 15 simulated years, 2086-2100, are expressed as the percentage change: [(mean scenario with evolution - mean scenario without evolution)/mean scenario with evolution] x 100. A positive number indicates that evolution increases the value of the population output. Conversely, a negative number indicates that the model output is higher in the hypothetical non-evolving population.

| **Population outputs** | **35%** | **50%** | **65%** |
| --- | --- | --- | --- |
| Density total (trout/ha) | -1.6 | -2.1 | 1.4 |
| Density age-0 | -1.8 | -2.9 | 0.4 |
| Density age-1 | -3.6 | -2.3 | 1.5 |
| Density age-2 | 8.8 | 16.2 | 32.0 |
| Density age-3Plus | 19.5 | 44.1 | 63.7 |
| Biomass total (kg/ha) | 1.3 | 2.7 | 4.4 |
| Biomass age-0 | 0.7 | 1.4 | 1.0 |
| Biomass age-1 | 1.3 | 2.1 | 2.5 |
| Biomass age-2 | 1.5 | 8.5 | 16.4 |
| Biomass age-3Plus | 2.9 | 29.6 | 47.0 |
| Ratio adults to juveniles (unitless) | 4.0 | 14.1 | 18.5 |
| Density spawners (trout/ha) | 16.9 | 28.6 | 34.5 |
| Number eggs (eggs/ha) | 8.2 | 9.9 | 11.3 |
